# High-throughput end-to-end aphid honeydew excretion behavior recognition method based on rapid adaptive motion-feature fusion

**DOI:** 10.3389/fpls.2025.1609222

**Published:** 2025-07-07

**Authors:** Zhongqiang Song, Jiahao Shen, Qiaoyi Liu, Wanyue Zhang, Ziqian Ren, Kaiwen Yang, Xinle Li, Jialei Liu, Fengming Yan, Wenqiang Li, Yuqing Xing, Lili Wu

**Affiliations:** ^1^ College of Science, Henan Agricultural University, Zhengzhou, Henan, China; ^2^ College of Computing, City University of Hong Kong, Hong Kong SAR, China; ^3^ College of Plant Protection, Henan Agricultural University, Zhengzhou, Henan, China

**Keywords:** honeydew excretion detection, aphid behavior recognition, rapid adaptive motion feature fusion, Kolmogorov-Arnold networks, RT-DETR-RK50

## Abstract

**Introduction:**

Aphids are significant agricultural pests and vectors of plant viruses. Their Honeydew Excretion(HE) behavior holds critical importance for investigating feeding activities and evaluating plant resistance levels. Addressing the challenges of suboptimal efficiency, inadequate real-time capability, and cumbersome operational procedures inherent in conventional manual and chemical detection methodologies, this research introduces an end-to-end multi-target behavior detection framework. This framework integrates spatiotemporal motion features with deep learning architectures to enhance detection accuracy and operational efficacy.

**Methods:**

This study established the first fine-grained dataset encompassing aphid Crawling Locomotion(CL), Leg Flicking(LF), and HE behaviors, offering standardized samples for algorithm training. A rapid adaptive motion feature fusion algorithm was developed to accurately extract high-granularity spatiotemporal motion features. Simultaneously, the RT-DETR detection model underwent deep optimization: a spline-based adaptive nonlinear activation function was introduced, and the Kolmogorov-Arnold network was integrated into the deep feature stage of the ResNet50 backbone network to form the RK50 module. These modifications enhanced the model’s capability to capture complex spatial relationships and subtle features.

**Results and discussion:**

Experimental results demonstrated that the proposed framework achieved an average precision of 85.9%. Compared with the model excluding the RK50 module, the mAP50 improved by 2.9%, and its performance in detecting small-target honeydew significantly surpassed mainstream algorithms. This study presents an innovative solution for automated monitoring of aphids’ fine-grained behaviors and provides a reference for insect behavior recognition research. The datasets, codes, and model weights were made available on GitHub (https://github.com/kuieless/RAMF-Aphid-Honeydew-Excretion-Behavior-Recognition).

## 1 Introduction

Aphids were globally recognized as significant phytophagous piercing-sucking pests that caused multiple forms of damage to agricultural and ecological systems. Their harmful effects were manifested through three primary mechanisms: direct feeding on phloem sap that impeded plant growth and development, secretion of honeydew rich in sugars and amino acids that induced sooty mold disease, and efficient transmission of plant viruses (Guerrieri and Digilio, 2008; Singh and Singh, 2016). In sorghum-growing regions across the United States, Mexico, and South America, aphid infestations resulted in 50%-100% crop losses (Thudi et al., 2024). Due to their exceptionally high reproductive capacity, polyphagy, and multiple adaptive traits, aphids emerged as one of the most destructive pest categories in global agricultural production.

The significant threat aphids posed to agriculture rendered in-depth research into their behavior of substantial scientific significance and practical value. Aphid feeding behavior not only reflected insect adaptation to host plants but also served as a critical indicator for evaluating plant resistance mechanisms (Zhang et al., 2024). Accurate monitoring of aphid feeding behavior was found to provide important guidance for pest management, resistance breeding, and crop protection. In particular, HE is directly related to feeding behavior, and HE has a very prominent visual feature. Detecting HE behavior is considered to be an ideal way to indirectly monitor aphid feeding status and assess plant resistance levels. Breeding aphid-resistant crop varieties is considered to be one of the more sustainable strategies for controlling these pests (Thudi et al., 2024; Zhang et al., 2024), and accurate monitoring and analysis of aphid-resistant behavior can provide critical technical support for the process of breeding aphid-resistant crop varieties, thus creating good conditions for sustainable agricultural development.

At present, to accurately monitor aphids’ feeding behavior, the main method is to rely on electropenetrogram technology, which records the electric potential changes of insect stigma in plant tissues, so as to distinguish different behavior states of aphids (Tjallingii, 1978). EPG technology has been successfully used to reveal a variety of aphids’ behavior patterns. For example, the specific piercing behavior of phloem cells related to virus transmission (Jiménez et al., 2020), and the changes in aphids’ behavior after pathogen infection (Chen et al., 2018). Although the machine learning method has improved the efficiency of EPG data analysis (Willett et al., 2016), this technology still has some prominent shortcomings. Connecting insects to the wire on the instrument will restrict the natural behavior of insects, making them unable to move freely. Waveform analysis also requires experts to do tedious manual annotation. This is a process that takes time and is difficult to scale up (Tayyab et al., 2024). Traditional methods of monitoring aphids’ HE behavior, such as manual visual counting or chemical analysis, also have problems of low efficiency and poor real-time performance, and cannot accurately capture this key behavior of aphids’ activity characteristics. These technical limitations make researchers want to explore new monitoring methods based on machine vision. These methods are touch-free and do not have invasive effects on the subjects.

Behavior recognition is an important area within machine vision, requiring not only the localization of target individuals but also a deep understanding and analysis of their behavioral patterns (Manoukis and Collier, 2019). In recent years, the rapid development of deep learning technology has significantly driven advances in behavior recognition research. Architectures such as two-stream convolutional networks (Simonyan and Zisserman, 2014) and spatiotemporal fusion models (Feichtenhofer et al., 2016) have provided powerful technical support for behavior recognition in videos by effectively integrating appearance and motion information. These methods have been successfully applied across multiple domains, particularly in behavioral analysis of large targets such as cattle (Yang et al., 2025; Zhang et al., 2024), pigs (Zhang et al., 2024), sheep (Gu et al., 2023; Zhang et al., 2024b), poultry (Ahmed et al., 2024; Zhao et al., 2024), and fish (Yu et al., 2025; Hu et al., 2025; Zhao et al., 2024; Du et al., 2023), all achieving recognition accuracies exceeding 90%.

In the field of insect behavior recognition, several pioneering studies have made progress. Qiao et al. (2018) created a system for automatic detection of fruit fly grooming behavior, using K-nearest neighbor algorithms to achieve high-precision classification of three behaviors: grooming, movement, and rest. Professor Zhan’s team conducted in-depth research on grooming behavior recognition in *Bactrocera minax*, with Zhang et al (2024a) proposing a grooming behavior detection method based on spatiotemporal context and CNN, and Zhan et al. (2021) implementing tephritid key point tracking and grooming behavior recognition using DeepLabCut. Xiong et al. (2024) applied computer vision technology to identify and track the regurgitation behavior of fruit flies, achieving 96.3% recognition accuracy using the I3D model.

However, research on behavior recognition of small insects, especially piercing-sucking pests like aphids, remains in its early stages and faces a series of unique challenges. Compared to large animals, aphids are minuscule (minute targets) with rapid movements, concealed behaviors, and indistinct features (Noldus et al., 2002), making conventional visual monitoring methods inadequate for precisely capturing their behavioral details. In particular, the feeding behavior of aphids is almost completely invisible as their stylets penetrate plant tissues, requiring indirect characterization through observable behaviors such as HE. Additionally, aphids’ semi-transparent nature and similarity to plant background colors make target segmentation exceptionally difficult, while their aggregation characteristics and adhesion between individuals further increase the complexity of behavior recognition. Existing insect behavior recognition research is difficult to directly apply to agricultural pests such as aphids, mainly facing the following challenges: First, differences in research subjects and environments exist. Existing research (Qiao et al. (2018); Zhang et al. (2020); Zhan et al. (2021); Xiong et al. (2024)) focuses on high-contrast laboratory model organisms like fruit flies, while aphids are smaller and have semi-transparent characteristics that make them difficult to distinguish from plant backgrounds. Second, the complexity of analysis scenarios is an issue. Traditional research typically analyzes individual insect behavior under ideal conditions such as petri dishes (Zhang et al. (2020)), while aphids in actual agricultural scenarios often exist in dense colonies on tobacco, cotton, or wheat leaves, with complex backgrounds and frequent overlap between individuals. Third, the specificity of behavior recognition targets poses a challenge. Existing research often focuses on behaviors that affect the insects themselves, such as grooming behavior, and rarely focuses on behaviors with ecological and agricultural significance, especially HE behavior that directly characterizes the feeding status of aphids, which is not only subtle and transient but also lacks specialized recognition methods. Additionally, existing technical methods themselves have serious limitations. Frame difference-based motion feature extraction research suffers from slow processing speed, high motion feature noise (Zhang et al. (2020)).

Furthermore, significant differences between feature maps and original RGB video frames captured by the camera leading to loss of key spatial information. Consequently, two-stage detection processes have poor real-time performance, unable to meet high-throughput detection requirements. Therefore, there is currently no specialized multi-object high-throughput end-to-end behavior detection platform for aphid populations. These special challenges necessitate the development of entirely new technical solutions for aphid behavior monitoring to adapt to the practical needs of agricultural pest monitoring.

Based on the research status and technical challenges outlined above, this study proposed an innovative solution for aphid behavior recognition. Key innovations included: (1) Establishment of the first aphid behavior dataset encompassing three characteristic behavioral patterns CL, LF, and HE, laying the data foundation for automated aphid behavior analysis; (2) Development of a Rapid Adaptive Motion Feature fusion (RAMF) method that achieved real-time processing of high-resolution videos through innovative temporal weighting mechanisms, adaptive threshold design, and parallel computing optimization, effectively extracting motion features of minute targets; (3) Design of an enhanced object detection architecture, RT-DETR-RK50, which integrated KAN modules into deep stages of the ResNet-50 backbone network, introducing spline-based adaptive nonlinear activation functions to enhance the network’s capacity to capture complex spatial relationships; (4) The proposed high-throughput end-to-end behavior recognition platform is not only applicable to aphids but can also be extended to the behavior recognition of similar insects such as whiteflies, providing a novel technical pathway for real-time pest behavior monitoring.

The high-throughput end-to-end real-time visual recognition framework established in this study not only solved the technical challenges of aphid behavior monitoring but also innovatively characterized aphid feeding status and intensity indirectly through HE behavior recognition, providing a new observational window for pest-plant interaction research. By automatically recognizing key aphid behaviors including CL, LF, and HE, particularly using HE as an indicative behavior of feeding activity, researchers can precisely evaluate plant resistance levels and predict pest damage, thereby optimizing control strategies. This technological advancement contributes to promoting integrated pest management practices, reducing pesticide dependence, and achieving more environmentally friendly agricultural production. As an efficient phenotypic analysis tool, this method can accelerate resistant variety breeding processes, shortening breeding cycles.

## 2 Materials and methods

### 2.1 Original materials

**Figure 1:**
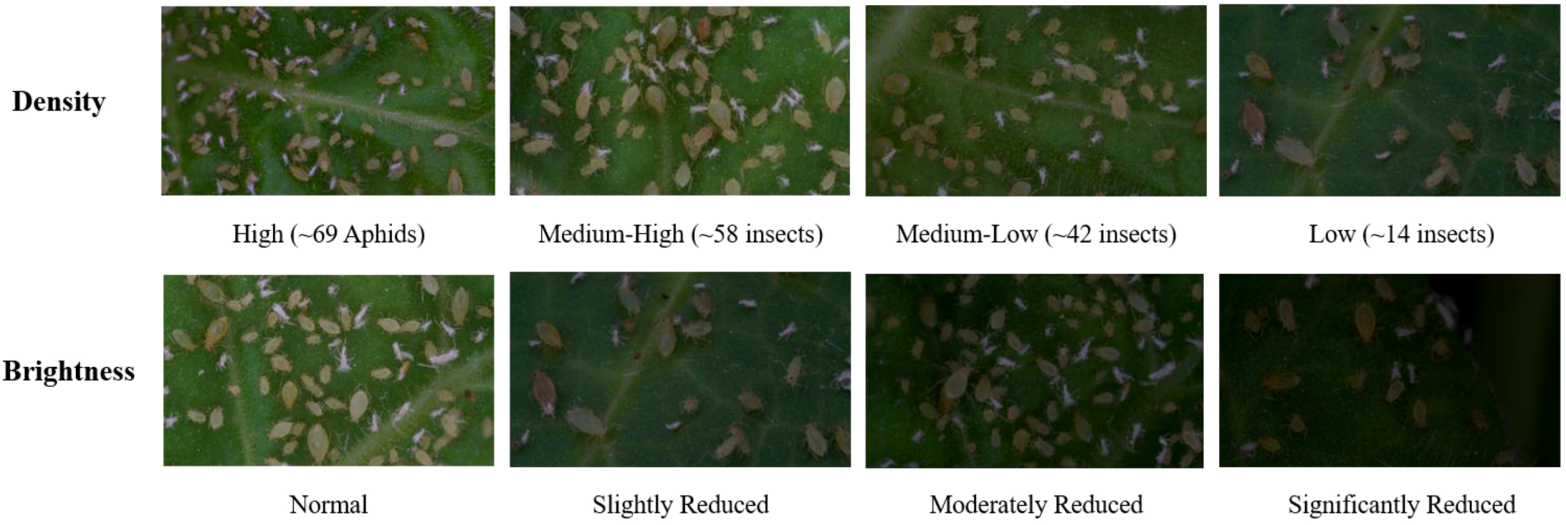
Dataset images of different population densities and light intensities.

Current aphid research primarily relies on field collection, counting, and detection methods, with relatively limited focus on behavioral studies. Greenhouse cultivation and recording of aphids present significant challenges. This study has established the first comprehensive aphid behavior dataset to address this gap. The dataset encompasses various population densities and different light intensities, as illustrated in **Figure 1**.

For the experiments, *Myzus persicae* were maintained in isolation on healthy tobacco plants under greenhouse conditions. The rearing environment was strictly controlled at 25°C, 70% relative humidity, and a 14:10 (L:D) photoperiod. Only wingless adult aphids were selected for the experiments. Tobacco plants (Zhongyan No. 1) were cultivated in pots within an artificial climate chamber. Plants were watered with distilled water once every half-week and nutrient solution once every three weeks, with no pesticide applications. Plants at a uniform growth state (6–7 leaf stage) were selected for experimentation.

The experimental dataset was obtained at the modern greenhouse facility of Henan Agricultural University’s College of Plant Protection, spanning from August to December 2024. Standardized data acquisition was conducted using a high-resolution microscopic imaging system (Sony ILCE-7RM2 camera with Laowa 25mm f/2.8 ULTRA MACRO 2.5-5X lens, 1920×1080 pixel resolution, 30 fps sampling rate) under the same controlled conditions. All recordings were saved in color JPEG format to maintain visual detail while optimizing storage efficiency. All observation fields were carefully designed to encompass complete aphid activity zones while systematically including specimens at different developmental stages (nymphs and adults). The finalized time-series dataset comprises 32 independent experimental video groups, each corresponding to specific light intensity conditions, with individual group duration of 30 minutes and cumulative valid observation time reaching 16 hours.

### 2.2 RAMF: rapid adaptive motion-feature fusion

**Figure 2:**
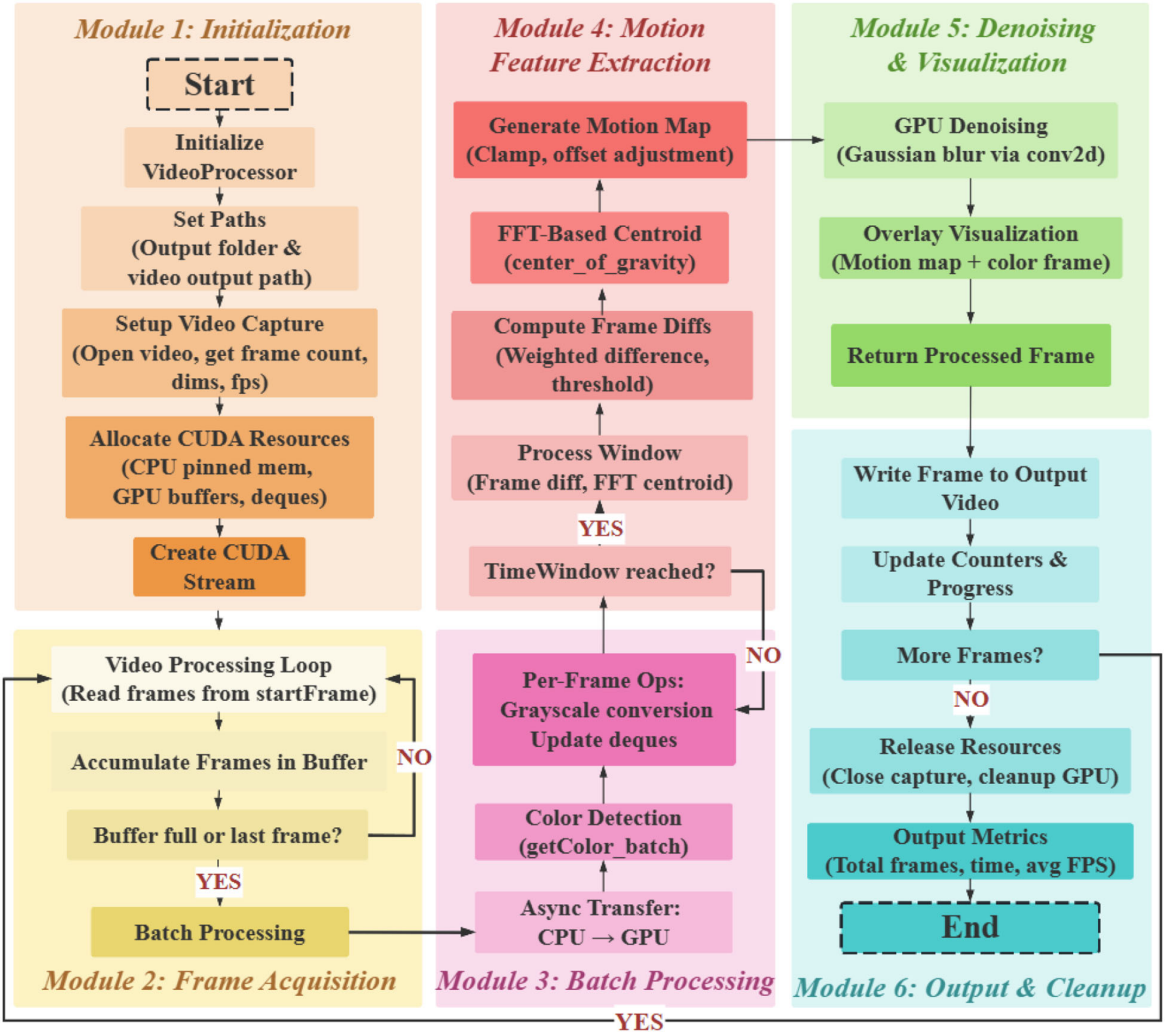
Flowchart of the RAMF framework.

To effectively extract motion feature information from aphids using frame differencing methods, which facilitates learning and classification by deep neural networks, a Rapid Adaptive Motion-Feature Fusion (RAMF) framework for moving object detection is proposed in this paper. This framework incorporates spatiotemporal feature adaptive fusion, as depicted in the schematic diagram in **Figure 2**.

The RAMF framework consists of six modules. The initialization (Module 1) sets up the video processing environment. Frame Acquisition (Module 2) reads and buffers frames. Batch Processing (Module 3) handles frames in batches. Motion Feature Extraction (Module 4), the core of the framework, generates motion maps and computes frame differences. Denoising and Visualization (Module 5) denoises the motion map and overlays it on the RGB frame. Output and Cleanup (Module 6) writes the processed video and releases resources. The framework iteratively processes video frames, using adaptive motion feature extraction to capture aphid motion characteristics for analysis.

**Figure 3:**
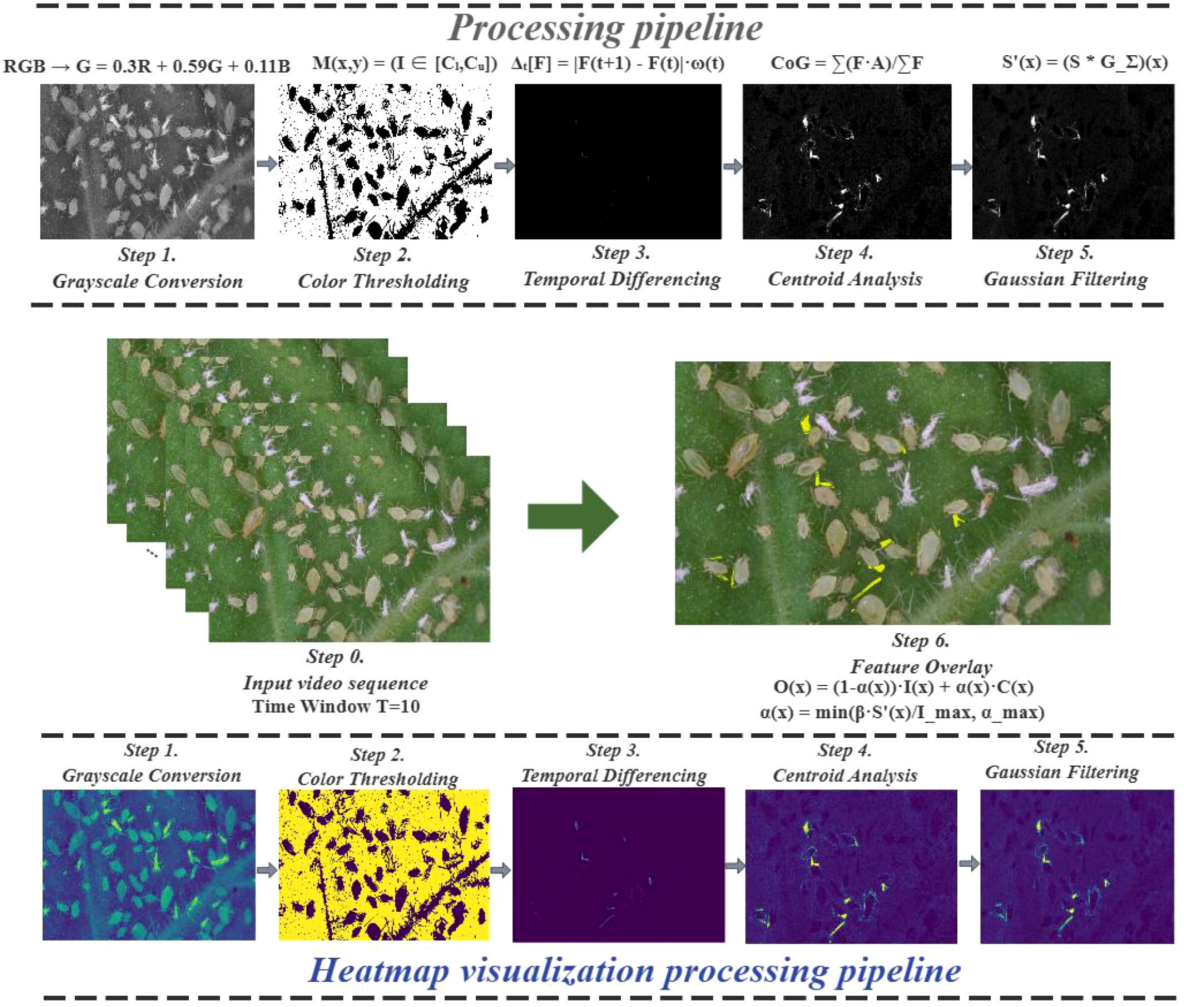
RAMF processing workflow and visualization diagram.

This fusion strategy preserves the morphological structure characteristics of the targets and enhances the temporal motion features through temporal weighting, effectively combining static and dynamic information. Compared to traditional frame difference methods, the proposed RAMF approach significantly suppresses noise interference and provides a more comprehensive feature description for object detection through three core technical innovations. First, adaptive temporal weighting is used for feature importance allocation (Equation 3). Second, statistical thresholding quantifies motion saliency (Equations 5, 6). Third, a self-adaptive adjustment mechanism is used for feature fusion coefficient adjustment (Equations 8, 9). The synergistic integration of static morphological features and dynamic motion characteristics significantly improves feature discrimination while maintaining information completeness. The processing pipeline and its corresponding heatmap visualization are presented in **Figure 3**.

#### 2.2.1 Temporal motion feature processing

Motion features can be detected using frame differencing (analyzing pixel value changes within a time window), which aids in behavior recognition (Ravbar et al., 2019). To accurately capture target motion characteristics, the RAMF method uses a sliding time window based on frame differencing to process consecutive video frames. The time window length impacts motion feature extraction quality larger windows are better for capturing low-frequency motion but may miss instantaneous changes, while smaller windows capture detailed features but are more noise-sensitive. Therefore, the window size must match the target motion’s frequency characteristics.

To determine the optimal time window length, this study conducted a systematic evaluation of a set of time window lengths T=5,7,9,11,13,15,17 (with 2-frame intervals). The frame difference of continuous video sequence F is analyzed, and its motion features are presented in a visual way. From the experimental results, the window length between 9 and 11 has reached the best balance state. This balance is reflected in that it can avoid the prominent motion tailing effect while ensuring the features have relatively high clarity. This paper selects their average $\bar{T}=10$ frames and takes it as the final window length.

Within the time window, the difference between adjacent frames is calculated using the following weighted approach, as shown in Equation 1:


(1) $$\Delta_{t}\;\left[F\right]=\Psi \left(F\left(t+1\right),F\left(t\right)\right)\cdot \omega \left(t\right)$$

In this formula, $\Delta_{t}\left[F\right]$ represents the weighted difference image of the video sequence F at time t , where F(t) refers to the image of the video sequence F at time t , Ψ(·,·) represents the difference operator, and ω(t) represents the time-weighted coefficient. Here, the difference operator is defined as the absolute difference of pixel intensities, as shown in Equation 2:


(2) Ψ(F(t+1),F(t))=|F(t+1)−F(t)|

In order to highlight the contribution of the most recent frame to the current motion state, the RAMF framework specifically designed an exponentially increasing weight allocation scheme to reflect the criticality of the most recent frame as shown in Equation 3.


(3) $$\omega \left(t\right)=\frac{exp\left(\frac{t}{\bar{T}-1}\right)-1}{exp\left(1\right)-1},t\in \left[1,\bar{T}-1\right]$$

In this case, t represents the frame index within the time window, while $\bar{T}$ represents the size of the time window. This weight design has some advantages. First of all, exponential growth can ensure that the weight of the most recent frame in the time window will be higher, which is consistent with the biological visual system’s relatively high sensitivity to recent information. Secondly, the normalization process can ensure the stability of the feature scale, which improves the robustness of the algorithm.

#### 2.2.2 Motion saliency computation

To express the larger motion of the target region, the RAMF method will start by summing the weighted frame difference in the time window, and then build the initial motion map ℳ , as shown in Equation 4.


(4) $$ℳ\left(x\right)=\sum_{t=1}^{\bar{T}-1}\Delta_{\text{t}}\left[F\right]\left(x\right)$$

Here, ℳ(x) refers to the motion map value at the place where pixel x=(x,y) . $\Delta_{t}\left[F\right]$ refers to the weighted difference value of the video sequence F at pixel x at time t . Because in the real scene, the background noise will cause interference, the RAMF framework proposed an adaptive threshold mechanism based on statistical characteristics to set, as shown in Equation 5.


(5) $$\theta =\mu_{ℳ}+\kappa \cdot \sigma_{ℳ}$$

where $\mu_{ℳ}$ and $\sigma_{ℳ}$ respectively represent the mean and standard deviation of the motion map ℳ , κ  is an adjustable parameter, its role is to control the sensitivity of the threshold. In this study, κ is set to 3, which corresponds to the 3 σ criterion, and according to this threshold, the generation of a motility graph S can be expressed in the following formula.

On the basis of this threshold, the generation process of the motility graph S can be expressed in Equation 6.


(6) $$S\left(x\right)=\left\{ \begin{array}{l} M(x),\;if\;M(x)>\theta \\ 0,\;otherwise \end{array}\right.$$

Through this thresholding mechanism, background noise can be effectively suppressed while highlighting regions containing genuine motion targets.

#### 2.2.3 Adaptive feature fusion based on motion intensity

To enhance the continuity of feature representation and reduce local noise, the RAMF approach applies Gaussian filtering to the saliency map S as shown in Equation 7:


(7) $$S^{\prime }\left(x\right)=\left(S*G_{\text{Σ}}\right)\left(x\right)=\int_{\Omega }S\left(u\right)\cdot G_{\text{Σ}}\left(x-u\right)du$$

where G represents the Gaussian kernel with covariance matrix Σ , ∗ represents the convolution operation, and Ω represents the image domain. In this study, the size of G is 7×7, and $\Sigma =\sigma^{2I}$ , where σ=1.5 , and I is the identity matrix.

To effectively highlight motion regions while preserving original image details, the RAMF method designs an adaptive feature fusion scheme based on weighted mixing as shown in Equation 8.


(8) O(x)=(1−α(x))·I(x)+α(x)·C(x)

where I represents the original image, C represents the color visualization of motion saliency (using the yellow channel in this study), and α(x) is the adaptive mixing coefficient.

The mixing coefficient α(x) is calculated through a motion intensity-based adaptive mechanism as shown in Equation 9.


(9) $$\alpha \left(x\right)=min\left(\beta \cdot \frac{S^{\prime }\left(x\right)}{J_{\max}},\alpha_{max}\right)$$

where β is a scaling factor used to adjust the influence of motion saliency on the fusion coefficient, $J_{\max}$ is the maximum value of image pixel intensity (e.g., 255), and αmax is the upper limit of the mixing coefficient to maintain the visibility of the original image information. In this study, β is set to 1.2, and $\alpha_{max}$ is set to 0.8.

This adaptive strategy achieves reasonable enhancement of motion features while ensuring the original image information is not excessively occluded by dynamically adjusting the mixing ratio. Specifically, regions with higher motion intensity receive higher mixing coefficients, with an upper limit of 0.8 to maintain the visibility of the original image information.

#### 2.2.4 Accelerated processing design

The time difference method is effective in motion detection, but its computational complexity increases exponentially with the increase in pixel resolution and the size of the time analysis window, which limits its applicability in real-time, large-scale scenarios, such as panoramic multi-object recognition scenarios that require full frame processing. In the past, the region of interest technique has been used to alleviate the problem by tailoring input frames into local areas and then performing calculations (Zhang et al., 2020; Ravbar et al., 2019), but the ROI-based approach is inherently less suitable for panoramic systems, where global scene analysis and multi-target tracking are critical parts.

Recent advances in computing hardware and parallel processing have addressed the limitations of traditional time differentials with the help of system acceleration strategies that enable Gpus acceleration, efficient memory management, and algorithm-level optimization on NVIDIA RTX 4090 Gpus. Under the condition of 1080p resolution and 10 frame time analysis window, RAMF method can achieve 45 frames per second motion feature extraction, which proves that real-time high-resolution motion analysis is feasible. Critical design principles enabling this performance include parallel execution of intensive image processing tasks using GPU tensor operations, efficient CPU-GPU data transfer via pinned memory technology, overlapping operations through asynchronous CUDA streams, minimized overhead through batch processing and pre-allocated GPU resources, and acceleration of critical tasks using specialized libraries for GPU parallel math operations.

### 2.3 Construction of aphid behavioral classification dataset

**Figure 4:**
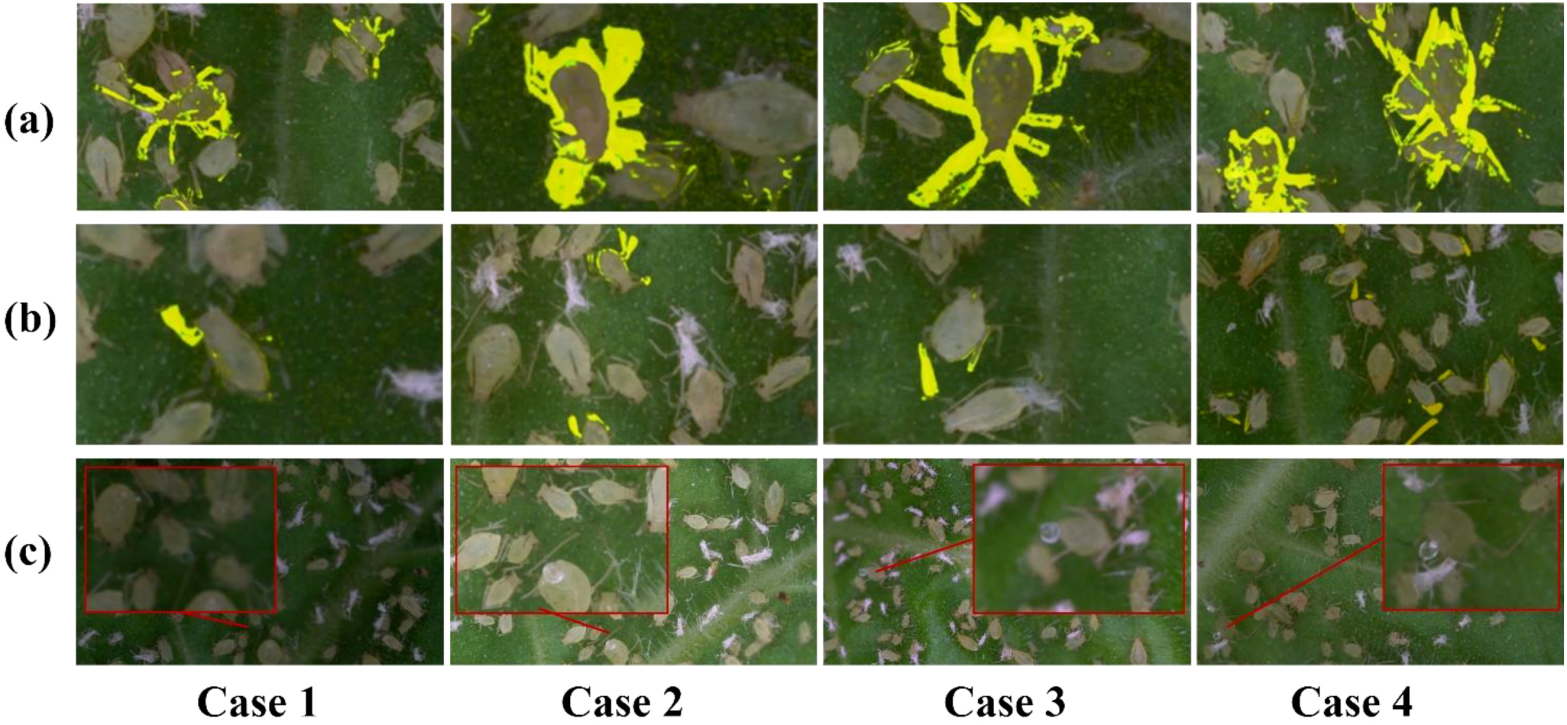
Aphid behavior classification: **(a)** crawling locomotion; **(b)** Leg Flicking; **(c)** Aphid Honeydew Excretion.

Following the motion feature extraction process described in Section 2.2, key frames were extracted from the original video at fixed intervals of 15 or 30 frames. The labeling annotation tool was utilized to annotate typical aphid behaviors in these key frames, including both position bounding boxes and behavior type labels. Three characteristic behavioral patterns were specifically documented: CL, LF, and HE. The visual features were associated with behavior labels based on the characteristics of the motion features extracted using the RAMF algorithm (based on frame differencing). Specifically, for the CL category, aphids exhibit whole-body movement, resulting in changes in overall pixel values and presenting as Whole-body contour optical flow. For LF, a periodic action involving only leg movement, only localized optical flow is observed. Finally, HE, the excretion of honeydew, is characterized by the optical feature of white, semi-transparent spheres. The distribution of valid behavioral samples is presented in **Figure 4**; **Table 1**.

**Table 1:** Aphid behavior dataset: behaviors and visual traits.

Behavior name	Number of labels		Visual features	Frame type
	Train	Val		
CL	2572	397	Whole-body contour optical flow.	Motion frames
LF	2078	866	Local optical flow of mid and hind legs.	Motion frames
HE	459	159	Translucent spherical microdroplets.	Original frames

A comprehensive aphid behavior analysis dataset was established through this process. This dataset encompasses various typical behavioral patterns of aphids under natural conditions, providing a robust data foundation for subsequent research on behavior recognition algorithms.

### 2.4 Research on object detection technology RT-DETR-RK50

#### 2.4.1 Overview of object detection techniques

Based on motion feature videos processed in Section 2.2, recognition algorithms must be applied for automated behavior identification. Traditional approaches include 3D convolutional neural networks (Xiong et al., 2024) and regions of interest (ROI) extraction for classification (Zhang et al., 2020; Ravbar et al., 2019). However, 3D CNNs are time-consuming with high annotation costs, while image classification involves complex processing with limited real time performance neither optimal for multi object aphid detection in natural environments.

Object detection techniques primarily divide into CNN and Transformer architectures. CNN detectors evolved from two-stage to single-stage models with both anchor-based and anchor-free paradigms (Alzubaidi et al., 2021), while Transformer detectors (DETRs) eliminate manually designed components for true end-to-end detection (Carion et al., 2020).

The YOLO series uses a single-stage design to achieve real-time performance (Redmon and Farhadi, 2018; Jocher et al., 2022, Jocher et al., 2023, Jocher and Qiu, 2024; Wang et al., 2024; Tian et al., 2025), however, most YOLO series methods rely on non-maximum suppression, which increases the computation latency, and YOLOv10 uses a dual allocation method to eliminate NMS (Wang et al., 2024), but there are still some challenges to achieve real end-to-end real-time performance with guaranteed accuracy. In contrast, DETR builds a very clean end-to-end pipeline, which has no anchor points and no NMS, but its convergence is slow and the computational complexity is quite high, which is a challenge for real-time applications, and improvements such as deformable detr (Zhu et al., 2020) and conditional DETR (Meng et al., 2021), while improving performance, can improve the performance of real-time applications. But the computing costs are still high.

RT-DETR represents a breakthrough real-time end-to-end detector (Zhao et al., 2024) that combines a backbone, hybrid encoder and transformer decoder with an auxiliary prediction head. Its hybrid encoder handles multi-scale features separately, applies self-attention at each scale, and uses an efficient pyramid structure for cross-scale fusion. The calculation cost is significantly reduced. RT-DETR’s IOU-aware query selection mechanism combines IoU constraints to select high-quality features as initial queries, which speeds up convergence and improves accuracy. It allows for flexible adjustment of reasoning speed with different decoder layers, without retraining. Compared to conventional detectors, RT-DETR eliminates the delays associated with NMS, while simplifying the pipeline and presenting an excellent speed-precision balance; it achieves faster reasoning at the same accuracy and higher accuracy at the same speed.

#### 2.4.2 RT-DETR-RK50: enhancing backbone architecture with KAN

Initially, RT-DETR uses the standard ResNet-50 (R50) backbone for feature extraction. This backbone uses a hierarchical structure that stacks bottleneck blocks at several stages. The traditional R50 backbone works well, but because it has a fixed core and a rigid activation function, RT-DETR is able to perform feature extraction. There are some inherent limitations in representational power. Such architectures have particularly significant limitations on the ability of models to capture complex spatial relationships and subtle feature representations, which are critical for accurate object detection tasks.

**Figure 5:**
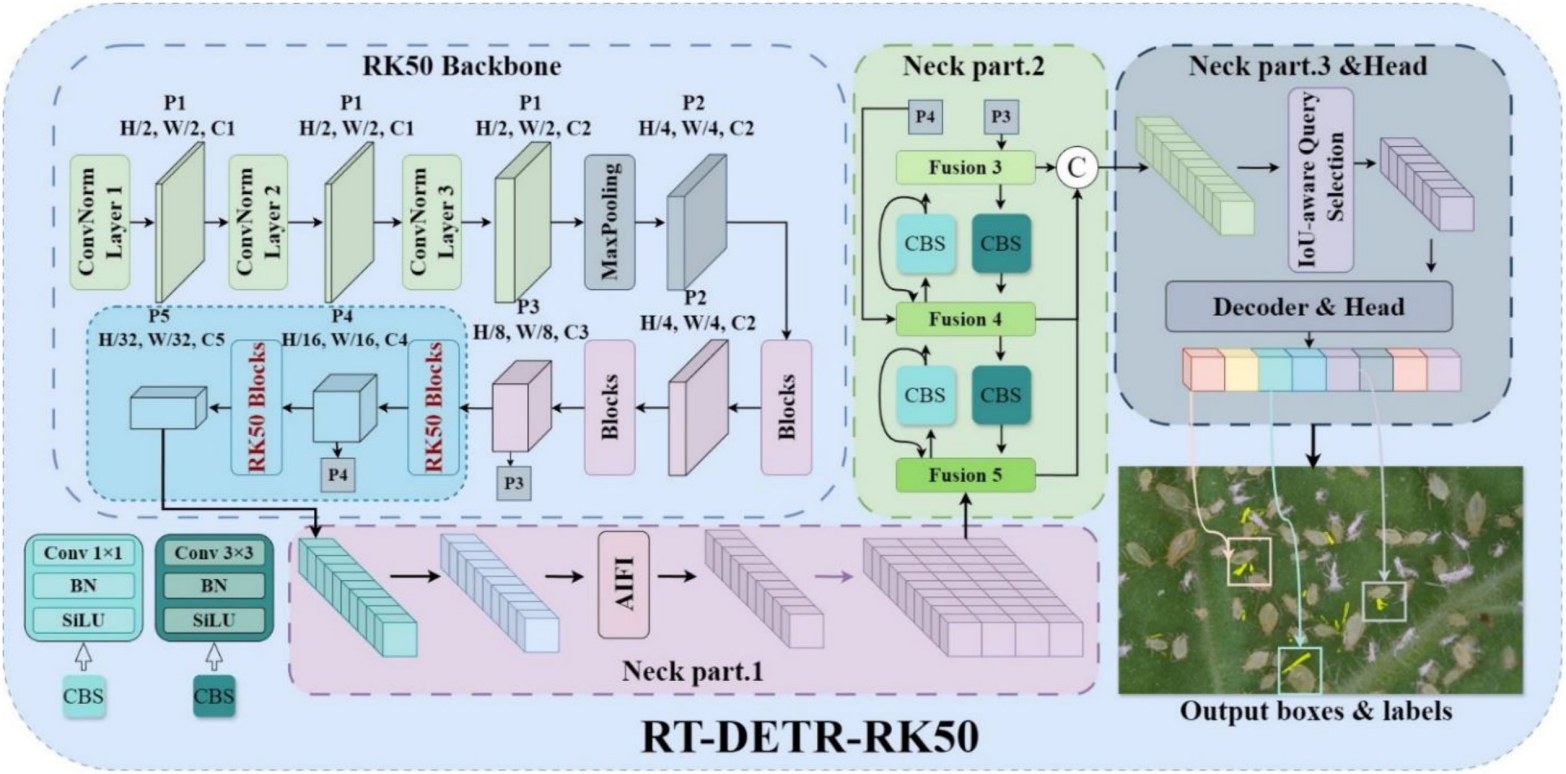
Architecture diagram of RT-DETR-RK50 network.

**Figure 6:**
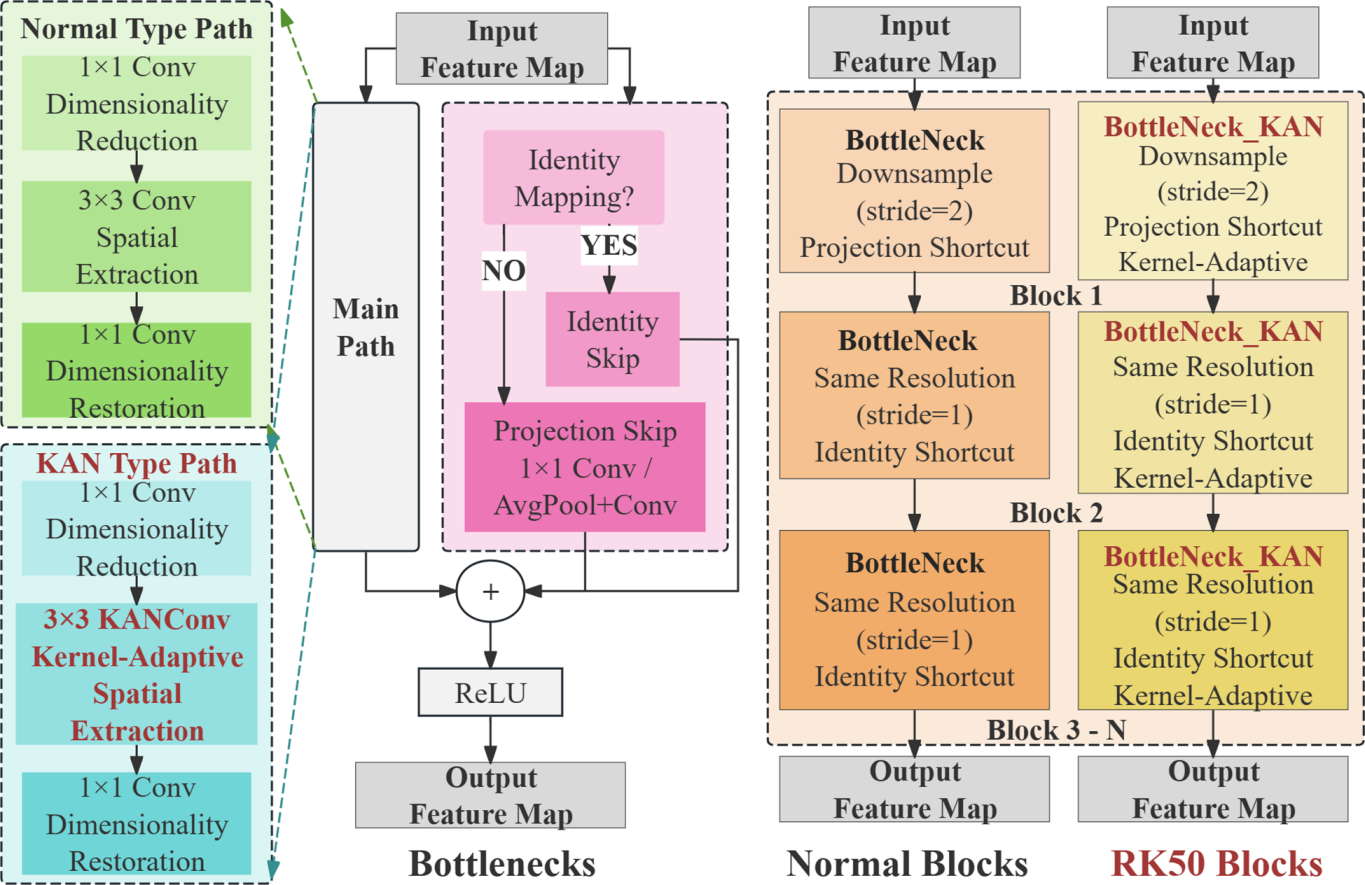
Network architecture diagrams of normal blocks and RK50 blocks.

To address these limitations, RT-DETR-RK50 is proposed as a new backbone network architecture that strategically uses the Kolmogorov-Arnold network, also known as KAN module. Integration into the key deeper stages of the ResNet-50 backbone (Liu et al., 2024). The approach is to replace the standard bottleneck blocks with KAN enhanced bottleneck modules, while maintaining the original architecture of the earlier stages while doing this replacement, thus specifically improving stages 4 and 5. This targeted enhancement is to focus computing resources on those areas that will bring the greatest benefit to feature representation. As shown in **Figure 5**. The concrete architecture of the Blocks and Bottleneck models is shown in **Figure 6**.

The key innovation in this structure lies in the implementation of kernel adaptivity through B-spline basis functions, enabling networks to learn more complex non-linear feature transformations beyond the traditional linear combinations typical of conventional convolutions (Drokin, 2024). As demonstrated in Drokin’s work, the deeper blocks in network architectures can be enhanced by utilizing BottleNeck_KAN (containing KAGNConv2DLayer) instead of standard BottleNeck modules. These KAN-enhanced bottleneck modules integrate spline-based activation functions capable of modeling arbitrary continuous functions, thus expanding the network’s representational capacity beyond traditional ReLU-based convolutions. This study implement adaptive nonlinearity that dynamically adjusts the data distribution. This adaptive nonlinearity promotes more flexible feature transformation, which is particularly useful in complex target boundaries and multiple detection scenarios. Due to the feature maps at stages P4 and P5 being reduced to 1/16 and 1/32 of the original image size, respectively, they exhibit a degree of abstraction and contain high-level semantic information. Consequently, the RK50 module is more appropriate for processing and enhancing these high-level features. Furthermore, the smaller feature map size at stage P5, coupled with a larger receptive field, provides richer global contextual information, which the RK50 module can effectively leverage to improve feature representation. Replacing the module at earlier stages may result in the loss of crucial detailed information or the introduction of excessive noise, potentially degrading performance. By selectively applying this enhancement only to the deep network layer responsible for high-level semantic features, rather than replacing all blocks, this study demonstrates an optimal balance between computational efficiency and detection accuracy, while significantly improving the effectiveness of feature extraction, a critical factor in detection performance.

This implementation uses a KAN-based convolution layer that combines traditional linear transformations with spline activation paths to create a more expressive feature modeling system. This design allows gradients to propagate through the network. It also improves the model’s ability to capture complex spatial patterns by integrating these advanced modules into the deep stages of the backbone. The rt-der-rk50 strategically improves characterization capabilities, which have the greatest impact on detection performance, and may result in a more efficient feature extraction pipeline without a significant increase in overall computational requirements.

The RT-DETR-RK50 backbone combines the structural efficiency of ResNet with the potential benefits of KAN technology in object detection applications, and it represents a very promising approach to real-time object detection architecture.

### 2.5 KAN Conv

#### 2.5.1 Kolmogorov-Arnold networks

Inspired by the Kolmogorov-Arnold representation theorem, Kolmogorov-Arnold networks represent a new architectural paradigm in deep learning. Unlike traditional multilayer perceptrons, which use fixed activation functions on nodes, KANs uses the learnable activation function on the edge, and replaces the traditional weight parameter with a single variable function parameterized to a spline.

The Kolmogorov-Arnold representation theorem states that any multivariate continuous function can be decomposed as shown in Equation 10:


(10) $$f\left(x\right)=f\left(x_{1},\ldots ,x_{n}\right)=\sum_{q=1}^{2n+1}\Phi_{q}\left(\sum_{p=1}^{n}\phi_{q,p}\left(x_{p}\right)\right)$$

where $\phi_{q,p}:\left[0,1\right]\to \mathbb{R}$ and $\Phi_{q}:\mathbb{R}\to \mathbb{R}$ are univariate functions.

KANs generalize this theorem by implementing network architectures of arbitrary widths and depths. A KAN layer with $n_{in}$ -dimensional inputs and $n_{out}$ -dimensional outputs is defined as a matrix of 1D functions, as shown in Equation 11:


(11) $$\Phi =\left\{\phi_{q,p}\right\},p=1,2,\ldots ,n_{in},q=1,2,\ldots ,n_{out}$$

For a KAN with l layers, the forward computation proceeds as shown in Equation 12:


(12) $$x_{l+1}=\Phi_{l}\left(x_{l}\right)$$

where each component of $x_{l+1}$ is computed as shown in Equation 13:


(13) $$x_{l+1,j}=\sum_{i=1}^{n_{l}}\phi_{l,j,i}\left(x_{l,i}\right)$$

The activation functions $\phi_{l,j,i}$ are parametrized as B-splines, as shown in Equation 14:


(14) $$\phi \left(x\right)=w_{b}b\left(x\right)+w_{s}\sum_{i}c_{i}B_{i}\left(x\right)$$

In this formula, b(x) is the basis function, under normal circumstances this basis function is the sigmoid linear unit, $B_{i}\left(x\right)$ is the B-spline basis function, $c_{i}$ is the coefficient that can be trained, this formula allows KANs to effectively learn the composition structure, but also can learn the function of one variable, This makes the model better than MLP in terms of accuracy and interpretability, especially for tasks that require complex functional approximations.

#### 2.5.2 KAN convolutional layers

**Figure 7:**
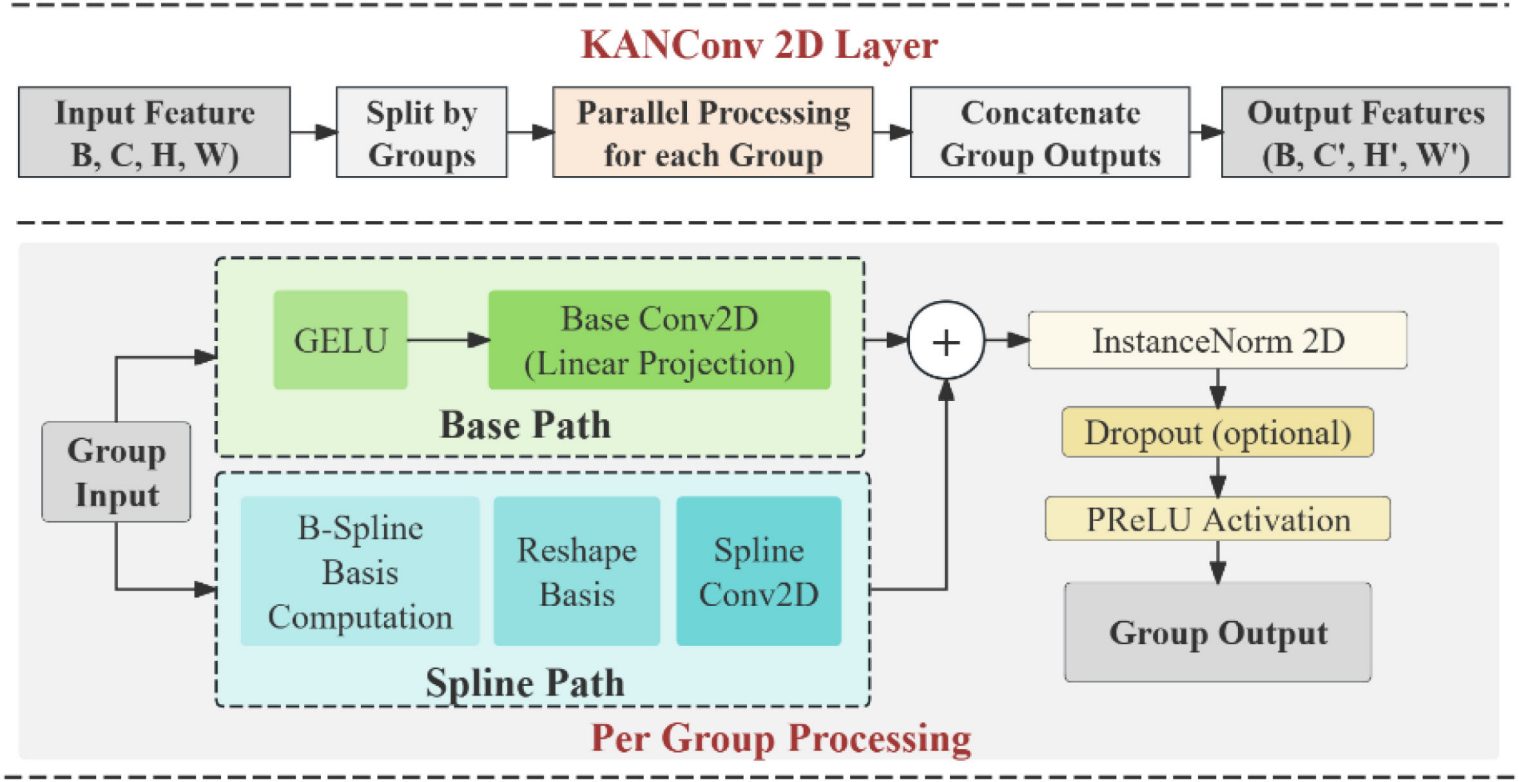
Network model diagram of KAN conv.


In fact, the principle of KAN can be extended to convolutional neural networks to form KAN convolutional layer, also known as KANConv. As can be seen from **Figure 7**, the architecture of KANConv2D layer is different from that of standard convolutional layer, which first applies fixed kernel operations and then performs nonlinear activation. KANConv integrates learnable activation functions directly into convolution operations. The KANConv layer operates by breaking the convolution into two parallel paths, one is the basic path, which applies the normal convolution operation to the input transformed by the basic activation function, and the other is the spline path, which applies the B-spline basis transformation to the input before convolution. The output of the KANConv layer of a single group can be expressed as shown in Equation 15:

(15) $$y=PreLU\left(Norm\left(Conv_{base}\left(g\left(x\right)\right)+Conv_{spline}\left(B\left(x\right)\right)\right)\right)$$

In this formula, g(·) represents the basis activation function, B(x) refers to the spline basis transformation, Norm is the normalization function, KANConv relies on the combination of learnable activation functions, improve the ability to approximate the function, compared with the fixed activation function. KANConv can more effectively model complex patterns present in the data. Spline-based approaches can adapt and learn feature transformations for specific data distributions on their own, rather than relying on pre-defined activation functions. This implementation also supports group convolution, which allows learning different activation functions for different feature sets, thus improving the representation of the model. dropout specifically designed for convolution dimensions provides efficient regularization during training. In essence, KANConv leverages the mathematical foundations of KANs to enhance the expressive power of convolutional operations while maintaining computational efficiency. This approach particularly benefits applications requiring complex function approximation in spatial or temporal domains, such as image processing, video analysis, and signal processing tasks.

### 2.6 Cross-frame processing for aphid honeydew excretion detection

Aphid honeydew excretion behavior serves as a key indicator of population vitality, characterized by small-amplitude movements that are difficult to capture. Although frame differencing successfully extracts excellent motion features, the optical characteristics of aphid honeydew are often obscured by overlaid optical flow, leading to difficulties in accurate identification under composite features. A simple solution strategy is to adopt multi-model training, specifically training RGB mode to identify honeydew and motion feature mode to recognize other behaviors, then merging the results. While this ensures high detection rates, it increases model complexity and reduces real-time performance.

#### 2.6.1 Cross-frame detection method

To address these issues, this study proposes a motion-original video cross-frame detection method. This approach processes only odd-numbered frames during the motion feature extraction phase, enabling detection of motion-based behaviors in odd frames while identifying key excretion targets (honeydew) in even frames. This method offers the following advantages: first, it ensures detection accuracy without requiring multiple models; second, it reduces computational resource consumption, as motion information differences between adjacent frames are minimal, allowing selective processing to decrease computational overhead by approximately 50% while maintaining information integrity.

#### 2.6.2 Post-processing algorithm and behavioral analysis strategy

Cross-frame processing causes detection results to exhibit a “flickering” phenomenon. To address this issue, based on temporal continuity assumptions, this study proposes a delayed interpolation improved post-processing algorithm: if the same behavior is detected in consecutive motion frames (such as the first and third frames), it is reasonable to infer that the intermediate original video frame (second frame) also contains this behavior. By extending single-frame detection results by one frame, the flickering problem is effectively eliminated.

Furthermore, aphid excretion behavior presents as multimodal features, divisible into static honeydew excreting (high-reflectivity droplets) and dynamic kicking actions (periodic motion trajectories). This study addresses the spatiotemporal heterogeneity by proposing a hierarchical three-phase analysis strategy. This strategy breaks down excretion behavior into three distinct phases: the first phase involves LF detection to identify high-frequency pre-excretion movement; the second phase involves honeydew identification to detect physical evidence of metabolic product generation in the form of single droplets; and the third phase involves composite motion assessment to determine honeydew excretion through coordinated observation. In practical detection, if both LF and honeydew appear in adjacent frames within a time window, they are identified as composite motion and labeled as “honeydew excreting,” thereby establishing a more precise vitality assessment model.

### 2.7 Real-time end-to-end behavior detection platform

Frame differencing methods play a vital role in behavior recognition and video analysis research. Traditional approaches typically followed a two-stage process: first extracting features to obtain feature videos or images, then feeding these features into algorithms for recognition or classification (Zhang et al., 2020). However, this approach faced two major challenges: insufficient real-time processing speed for feature extraction and substantial storage resources required for feature images, which significantly limited the practical application of frame differencing methods.

The improved frame differencing method RAMF proposed in this research achieved a processing speed of 45fps, enabling real-time processing of high-resolution (1080p) videos. Combined with the enhanced RT-DETR-RK50 detector, the input stage was optimized through streamlined processing, directly feeding extracted motion features into the detector seamlessly. This implementation realized a complete real-time end-to-end processing pipeline from video input to behavior detection output, effectively eliminating the need for intermediate data storage and improving overall system efficiency (with real-time processing speeds of 23.7fps on RTX3090 and 31fps on RTX4090 at 1080p resolution).

To achieve this goal, a comprehensive acceleration framework was implemented that employed a multi-threaded parallel pipeline architecture, decoupling the traditionally sequential stages of video processing. By implementing concurrent frame acquisition, feature extraction, and result rendering threads, the system effectively masked I/O latency and maximized computational resource utilization. GPU memory management was optimized through strategic pre-allocation techniques, reducing runtime fragmentation and minimizing the overhead of frequent memory operations. Through maintained persistent CUDA streams for parallel execution, the system achieved asynchronous computing where motion feature extraction proceeded simultaneously with object detection inference. The implementation leveraged batch processing optimizations, processing multiple frames concurrently rather than sequentially, with batch sizes up to 24 frames. Vectorized operations replaced traditional pixel-wise processing, further exploiting GPU parallelism for critical image processing operations. Model computation was enhanced through just-in-time compilation, converting the detection network into an optimized intermediate representation that substantially reduced interpreter overhead. This compilation was combined with mixed precision computation strategies while maintaining detection accuracy. The detailed pseudocode describing the comprehensive framework algorithm has been included in Appendix A.

Through the comprehensive application of these techniques, this research successfully constructed an efficient end-to-end real-time behavior recognition system, overcoming the limitations of traditional frame differencing methods with their cumbersome processing and poor real-time performance, providing a more practical solution for aphid HE video behavior analysis.

### 2.8 Experimental platform

The hardware configuration utilized an NVIDIA RTX 4090 GPU (24GB GDDR6X memory, AD102 architecture) for its powerful processing capabilities ideal for deep learning model training, supported by an Intel Xeon Gold 6430 (16-core) CPU for efficient data preprocessing and real-time augmentation. The software environment consisted of Python 3.12.3 with PyTorch 2.3.0 and Torchvision 0.18.0 (CUDA 12.1) to ensure framework-GPU architecture compatibility and optimized hardware utilization.

RT-DETR training configurations were systematically optimized: 200 training epochs without early stopping to fully realize model potential; AdamW optimizer balancing convergence speed and stability; 0.0001 initial learning rate suitable for stable Transformer training with 1.0 final learning rate factor maintaining learning capability; batch size 4 accommodating RT-DETR’s Transformer architecture memory requirements; and 0.0001 weight decay providing moderate regularization while preserving model expressivity. Loss balancing employed higher box loss weight (7.5) to enhance positional accuracy, lower classification loss weight (0.5) and moderate distribution focal loss weight (1.5) optimized for detection tasks. Training utilized 2000 warmup epochs with 0.8 momentum and 0.1 bias learning rate for early gradient stability. Cache mode was enabled to accelerate training, with 0.7 IoU evaluation threshold balancing precision and recall, and 18 parallel data loading threads optimizing data throughput. These parameters combined insights from YOLO implementations, DETR best practices, and hyperparameter optimization to precisely balance detection accuracy, training efficiency, and inference speed.

### 2.9 Evaluation metrics

#### 2.9.1 Object detection evaluation metrics

This study employs a multi-dimensional standardized metric system to quantitatively evaluate the performance of object detection algorithms. The core evaluation metrics include detection accuracy metrics, localization precision metrics, and real-time performance metrics.

For detection accuracy, the primary measurements are Precision and Recall. Precision reflects the accuracy of detection results, calculated as shown in Equation 16:


(16) $$P=\frac{TP}{\left(TP+FP\right)}$$

where TP (True Positive) represents the number of correctly detected objects, and FP (False Positive) represents the number of falsely detected objects.

Recall reflects the completeness of detection results, calculated as shown in Equation 17:


(17) $$R=\frac{TP}{\left(TP+FN\right)}$$

where FN (False Negative) represents the number of missed detections.

Localization precision is evaluated using IoU (Intersection over Union), calculated as shown in Equation 18:


(18) $$IoU=\frac{Area\;of\;Intersection}{Area\;of\;Union}$$

The overall detection performance is evaluated using mean Average Precision ( mAP ), specifically adopting mAP@0.5 as the primary performance metric, which represents the average precision across categories at an IoU threshold of 0.5, as shown in Equation 19:


(19) $$mAP=\frac{\sum_{i=1}^{n}AP}{n}$$

where n represents the total number of object categories.

In this paper, several key indicators are adopted to evaluate the complexity and real-time performance of the model. Among them, parameter counting quantifies trainable parameters in millions, which can reflect the storage demand and size of the model, and the computing burden is measured by GFLOPs. GFLOPs refers to billions of floating-point operations during forward propagation. Operational latency is calculated as the sum of pre-processing time ( tpre ), inference time ( tinf ), and post-processing time ( tpost ), as shown in Equation 20:


(20) Latency=tpre+tinf+tpost

This comprehensive evaluation framework balances the efficiency of the evaluation model to better balance the accuracy of the inspection with the utilization of computing resources and the feasibility of actual deployment.

## 3 Result and discussion

### 3.1 RAMF: comparison of different resolutions and time windows

**Figure 8:**
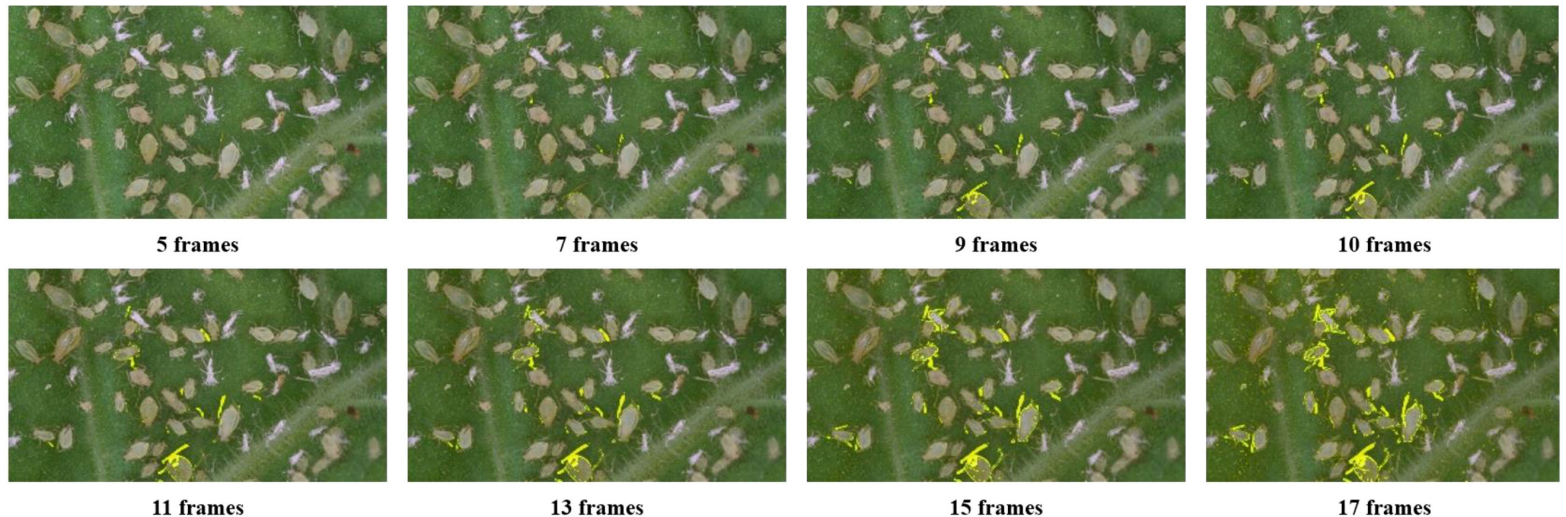
Visual representation of motion feature extraction across different temporal windows.

Resolution and time window size were identified as critical parameters in frame differencing methods that directly impacted the effectiveness of motion features and consequently influenced the accuracy of behavior recognition. In this study, a fixed 1080p resolution was maintained while the effects of various time window sizes were investigated. Results were shown in **Figure 8**.

The findings demonstrate that time window selection involves an important trade-off. Insufficient window sizes fail to capture complete motion patterns, while excessive window sizes accumulate multi-frame noise and cause motion features to blur, simultaneously increasing computational load. An optimal window size achieves balance between effectively capturing key behavioral characteristics and suppressing noise, ensuring motion feature distinctiveness and recognizability.

Through comparative analysis, it was determined that a time window size of 10 frames produced the best results, yielding motion feature images with superior highlight clarity and minimal trailing artifacts.

Regarding resolution considerations, higher resolutions involve more pixels in computation, resulting in increased computational complexity and reduced processing speed. However, before input to the object detection algorithm and resizing to 640 pixels, extraction of features at higher resolutions preserves more detailed characteristics. This preservation of fine-grained motion information ultimately contributes to improved detection performance despite the computational trade-offs.

As shown in **Table 2**, experimental results revealed a complex, non-linear relationship between time window size and processing speed (FPS) in video motion detection. At lower resolutions (480p, 720p), a moderate time window size (7–11 frames) initially improved performance, but larger windows led to performance degradation, potentially due to GPU parallelism efficiency and memory bandwidth saturation. Higher resolutions (1080p) exhibited more pronounced performance fluctuations, indicating that the algorithm was more susceptible to memory access patterns and cache utilization. Therefore, in optimizing video processing pipelines, the optimal time window size should be selected based on resolution, considering dynamic window adjustment and hardware characteristics. Future optimization directions include adaptive time window selection, optimizing memory access patterns, balancing workload, and conducting detailed performance analysis to fully leverage the benefits of GPU acceleration.

**Table 2:** RAMF processing speed at different resolutions and time windows.

Time window size (frame)	Processing speed (Fps)			GPU model	GPU power consumption	GPU memory usage
	1080p	720p	480p			
5	26.74	72.19	134.19	RTX3090	095.6W/350.0W	3911/24253MiB
7	31.84	53.33	162.85	RTX3090	113.1W/350.0W	11147/24253MiB
9	32.72	68.74	143.42	RTX3090	116.9W/350.0W	11147/24253MiB
10	21.54	78.16	137.85	RTX3090	117.1W/350.0W	11147/24253MiB
11	29.20	87.01	138.71	RTX3090	123.8W/350.0W	11147/24253MiB
13	25.19	84.02	131.69	RTX3090	125.0W/350.0W	11147/24253MiB
15	30.46	84.79	123.42	RTX3090	127.6W/350.0W	11147/24253MiB
17	29.76	81.18	118.48	RTX3090	131.5W/350.0W	11147/24253MiB
5	48.22	100.87	177.33	RTX4090	45.3W/450.0W	03643/24111MiB
7	44.91	103.06	216.84	RTX4090	51.7W/450.0W	11147/24111MiB
9	46.83	97.51	198.08	RTX4090	57.4W/450.0W	11147/24111MiB
**10**	**45.33**	**102.22**	**195.79**	**RTX4090**	**58.4W/450.0W**	**11147/24111MiB**
11	44.73	101.00	190.75	RTX4090	57.7W/450.0W	11147/24111MiB
13	43.43	95.48	187.96	RTX4090	57.7W/450.0W	11147/24111MiB
15	43.28	94.77	186.86	RTX4090	56.4W/450.0W	11147/24111MiB
17	46.77	92.96	183.76	RTX4090	63.2W/450.0W	11147/24111MiB

The final version presented in this article is in bold.

### 3.2 RT-DETR-RK50: ablation study on KAN module integration

To strike a balance between computational efficiency and detection performance, this study optimizes the deployment strategy of KAN modules within the rt-der-rk50 architecture. While KAN’s powerful adaptive activation and nonlinear modeling capabilities can significantly boost the performance of the ResNet-50 backbone, an excessive number of KAN modules may lead to a series of issues, including a sharp increase in computational complexity, increased optimization difficulty, overfitting risks, representational redundancy, chaotic feature levels, unstable gradient flow, and imbalanced resource utilization. To address these challenges, ablation experiments were systematically conducted to evaluate the impact of integrating varying numbers of KAN modules into the rt-der-rk50 architecture on detection performance. By comparing configurations such as RK50–1 and RK50-3, the aim is to determine the optimal number and distribution of KAN modules, thereby maximizing detection performance while ensuring computational efficiency.

#### 3.2.1 Feature representation optimization for RT-DETR-RK50 based on heatmaps

**Figure 9:**
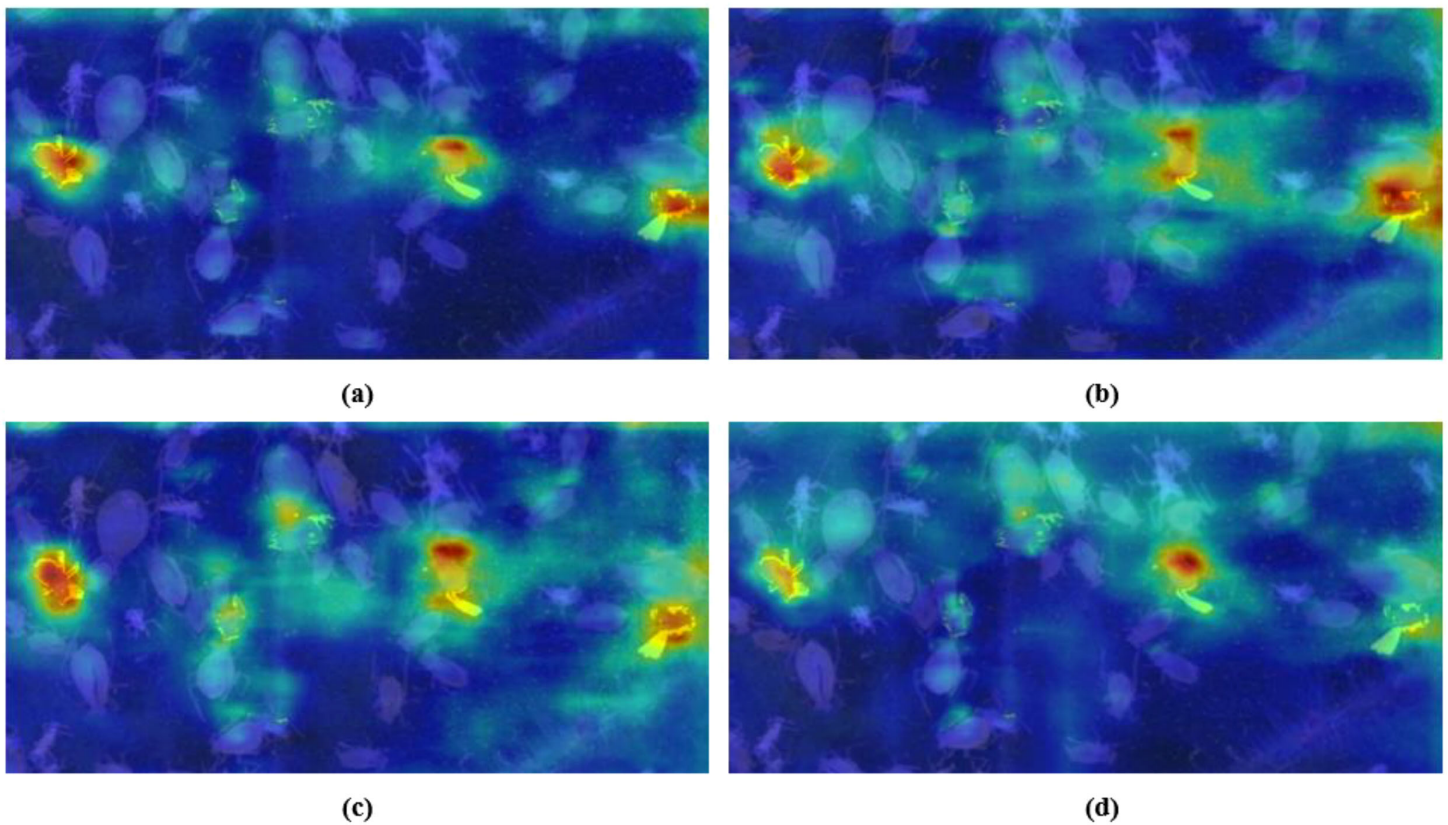
Feature activation heatmap comparison across RT-DETR-RK50 variants: **(a)** R50, **(b)** RK50-1, **(c)** RK50-2 (Ours), **(d)** RK50-3.

In order to verify the effectiveness of feature extraction in ablation research, this paper uses Grad-Cam ++ to carry out heat map visualization (Chattopadhay et al., 2018). There are specific parameters for heat map visualization here. The parameters are as follows: method is set to GradCAMPlusPlus, and layer 19 is set to 15th, 22nd and 25th respectively. Backward_type is null, confidence threshold is set to 0.2, and ratio is set to 1.0. It is clear from **Figure 9** that different RT-DETR backbone variants have different activation patterns, which also shows that there are significant differences in their feature representation capabilities.

The heatmap visualization in **Figure 8** reveals distinct activation characteristics across RT-DETR backbone variants. The baseline R50 model exhibits isolated hotspots with minimal inter-region connectivity, indicating effective but contextually limited feature identification. With progressive KAN block integration, significant transformations in feature representation emerge. RK50–1 demonstrates expanded activation fields with initial bridging between hotspots, suggesting enhanced spatial relationship modeling, albeit with suboptimal activation distribution. RK50–2 presents the most balanced activation topology, characterized by well-distributed hotspots with comprehensive connectivity and gradual transitions between high and medium activation regions. This pattern indicates sophisticated feature hierarchy development and optimal contextual integration. In contrast, RK50–3 exhibits more constrained activation patterns despite maintaining strong primary hotspots, suggesting potential representational redundancy and excessive specialization.

The quantitative metrics in **Table 3** corroborate these visual observations. RK50–2 achieves superior overall detection performance (0.849 mAP0.5) with remarkable consistency across behavioral categories, particularly excelling on the challenging Honeydew category (0.859 mAP0.5 versus 0.798 for R50). While RK50–3 shows marginal improvement on the CL dataset (0.862), it exhibits significant performance degradation on LF (0.693). Notably, RK50–2 maintains computational efficiency with only a 10.6% latency increase over the baseline (27.2ms versus 24.6ms), while delivering substantially improved detection capabilities.

**Table 3:** Results of ablation study on KAN module integration.

Model	mAP_0.5_ (CL)	mAP_0.5_ (LF)	mAP_0.5_ (HE)	F1 score (HE)	mAP_0.5_ (All)	Params (M)	GFLOPs (G)	Latency (Ms, bs=1)
R50	0.844	0.818	0.798	0.765	0.820	41.9	128.6	24.6 ± 0.2
RK50-1	0.854	0.809	0.715	0.734	0.793	70.2	129.6	28.4 ± 0.5
RK50-2	0.855	**0.832**	**0.859**	**0.847**	**0.849**	84.4	129.6	27.2 ± 0.7
RK50-3	**0.862**	0.820	0.769	0.810	0.817	86.7	129.6	32.6 ± 0.1

The best performers in each group are highlighted in bold.

Considering the limited number of training samples in the Honeydew class (only 459), relying solely on the mAP metric may not fully reflect the performance of the models on minority classes. This study employs the F1 Score metric, aiming to comprehensively consider Precision and Recall, thereby more comprehensively evaluating the performance of the models on imbalanced datasets. The F1 Score is the harmonic mean of Precision and Recall, effectively measuring the accuracy and completeness of the models in identifying minority classes. As can be seen from **Table 3**, the trend of F1 Scores for each model in the Honeydew class is generally consistent with mAP0.5 (Honeydew), indicating that the F1 Score and mAP metrics are correlated to some extent. Among them, the RK50–2 model achieved the highest F1 Score (0.847) in the Honeydew class, indicating that it has achieved a good balance between Precision and Recall, and can effectively identify the Honeydew class, reducing false negatives and false positives. In contrast, the R50 model has a lower F1 Score (0.765) in the Honeydew class, which may be more affected by class imbalance, resulting in some bias in its identification of the Honeydew class.

#### 3.2.2 Behavior classification accuracy analysis Based on confusion matrices

**Figure 10:**
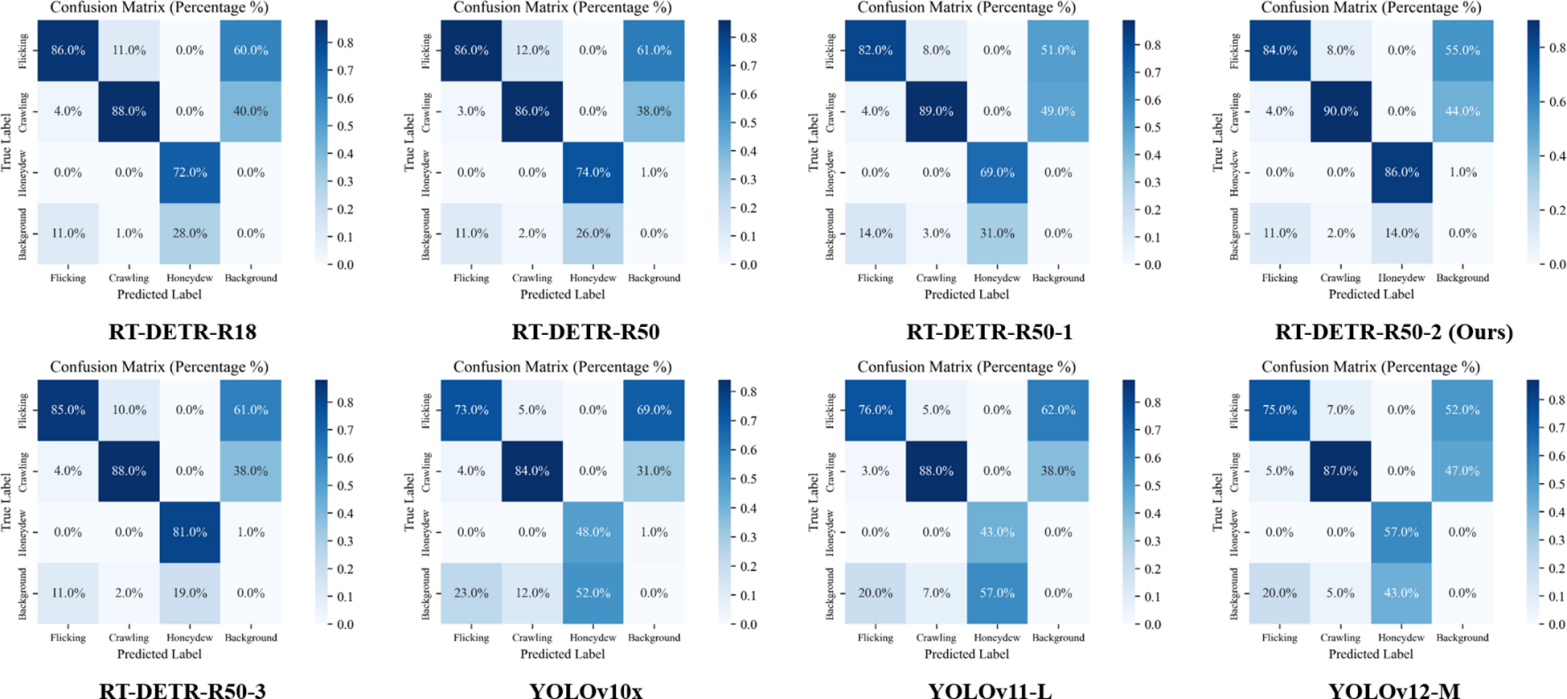
Confusion matrix comparison of detection models across different Aphid behavior categories.

The confusion matrices in **Figure 10** provide important insights into the classification performance of various detection models across three key aphid behaviors (CL, LF, and HE). The RT-DETR-R50–2 model demonstrates superior classification accuracy, with diagonal values significantly higher than other variants. Particularly in the Honeydew category, it achieves 86% accuracy, notably outperforming other RT-DETR backbone variants (R18: 72%; R50: 74%; R50-1: 69%; R50-3: 81%). The progressive improvement from R18 through various RK50 variants reveals systematic enhancement in behavior differentiation capabilities, with RK50–2 achieving optimal balance in reducing misclassification errors across all three behaviors, such as Honeydew being frequently misclassified as Background or LF being misidentified as CL in other models.

#### 3.2.3 Analysis of KAN module integration impact on model convergence

**Figure 11:**
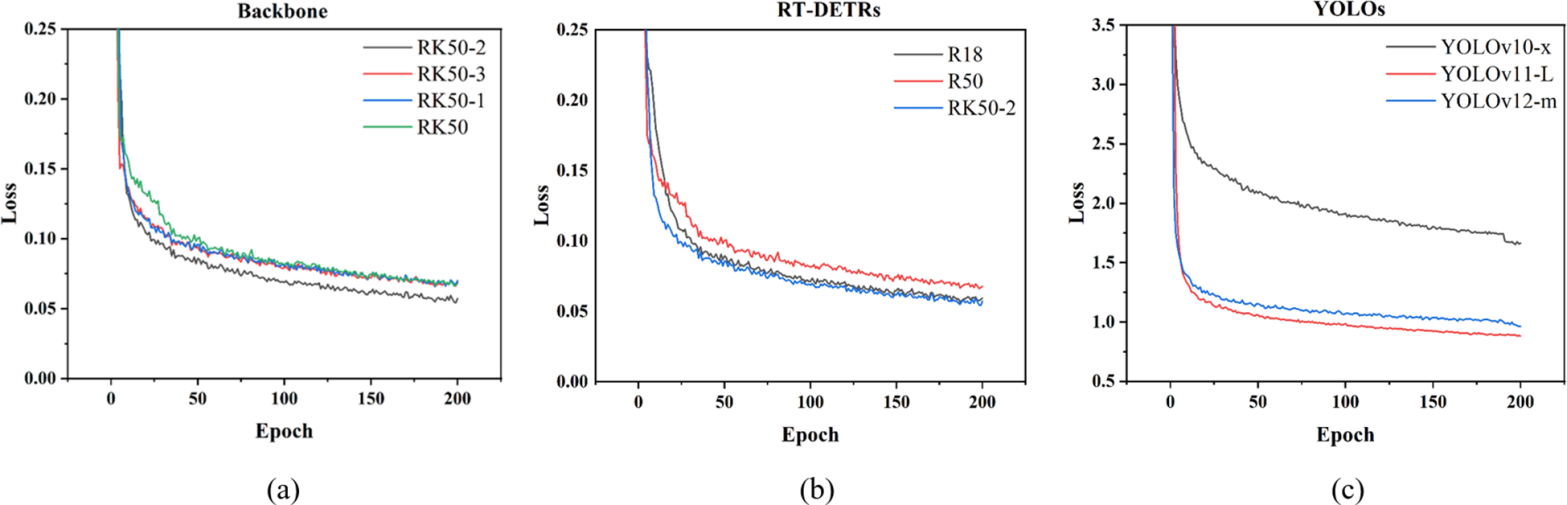
Comparison of training loss curves across different model architectures.

The loss curves in **Figure 11A** revealed RK50–2 as the optimal RT-DETR-RK50 variant, achieving the lowest steady-state loss (0.05) and fastest convergence, particularly in the first 50 epochs, while the baseline RK50 stabilized higher (0.07). RK50–1 showed limited improvement with its single KAN module, and notably, RK50–3 performed inferior to RK50–2 despite additional KAN modules, exhibiting oscillations in later training stages that validated the hypothesis of excessive nonlinearity causing optimization difficulties. RK50-2’s smooth curve indicated a more regular loss landscape and stable optimization process, with continuous improvement during the 75–150 epoch interval when other variants plateaued, confirming that two KAN modules in RT-DETR backbones represents an ideal balance between expressivity and training stability.

These findings validated the hypothesis regarding optimal KAN module deployment. The strategic integration of two KAN blocks achieved an ideal balance between enhanced representational capacity, effective spatial context modeling, and computational efficiency. The diminishing returns observed with additional KAN blocks confirmed concerns about excessive nonlinearity introducing optimization instabilities and function overfitting. The RT-DETR-RK50–2 configuration thus strategically leveraged adaptive nonlinearity to enhance detection performance without compromising inference efficiency.

### 3.3 RT-DETR-RK50: comparison of state-of-the-art methods

To ensure fair evaluation, all experiments were conducted in the same hardware environment equipped with an NVIDIA RTX 4090 GPU (SSD and Faster-RCNN were trained on RTX 3090 due to framework compatibility constraints) (Girshick, 2015; Liu et al., 2016). Representative state-of-the-art detection methods were selected for comparison, including YOLO series, DETRs family, Faster R-CNN, and SSD. As shown in **Table 3**, while the YOLO series excelled in detection speed and model efficiency, its detection accuracy was relatively limited. The proposed method achieved a significant improvement of 2.9% mAP over the baseline (RT-DETR-R50), attaining the best overall performance and validating its effectiveness. For practical deployment scenarios, a balance between accuracy and efficiency is crucial. RT-DETR-R18 emerged as the optimal choice within the DETRs family with 82.8% mAP and 15.9ms latency, while YOLOv12-M and YOLOv11-L demonstrated excellent accuracy-speed trade-offs in the YOLO series, with YOLOv12-M being particularly suitable for lightweight deployment scenarios.

**Figure 12:**
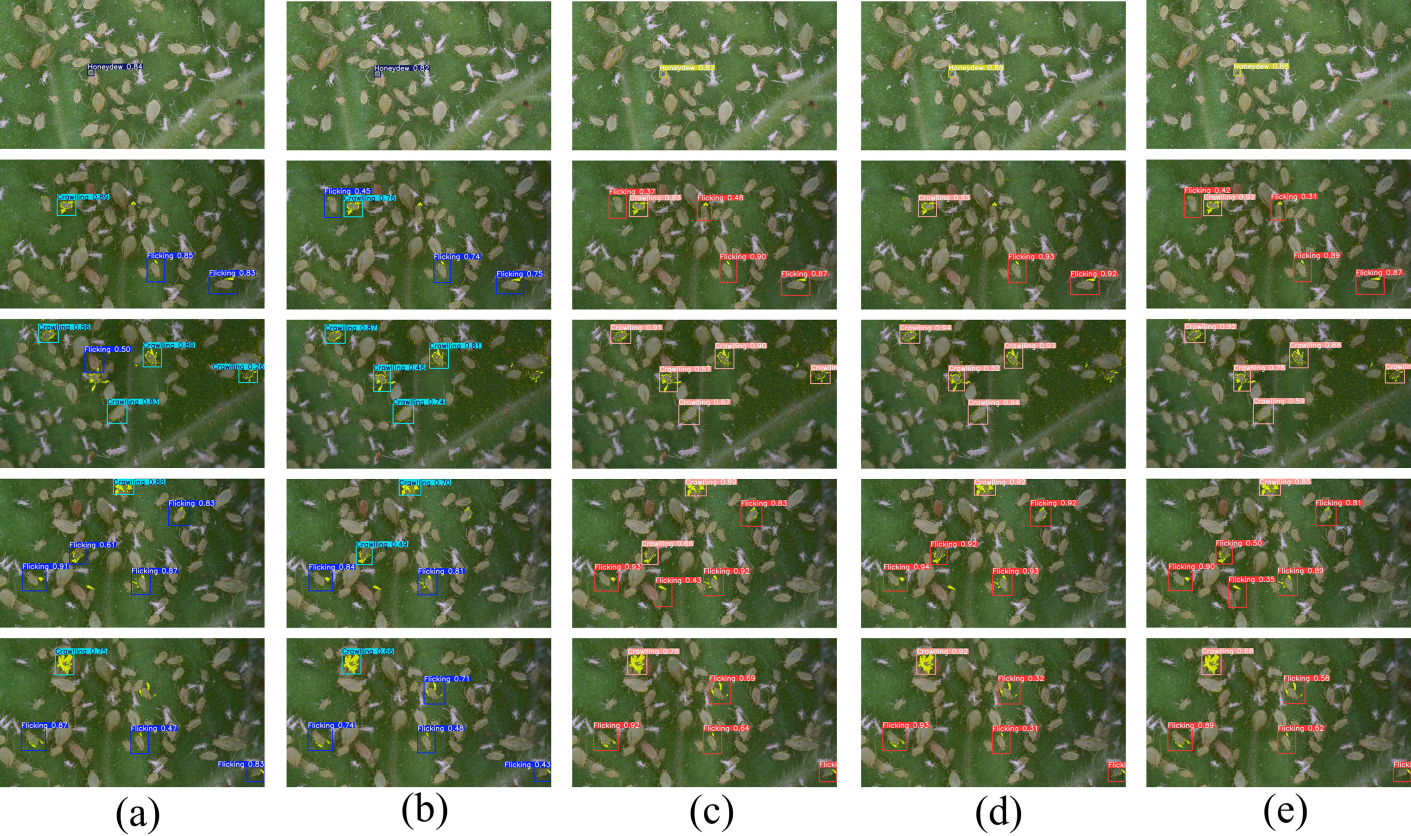
Detection results based on the model with the highest mAP50 metric.

**Figure 12** presented a visual comparison of detection results from five leading models on aphid behavior detection tasks. From left to right, the models were YOLOv11L, YOLOv12M, RT-DETR-R18, RT-DETR-R50, and the proposed RT-DETR-RK50–2 model. Through systematic comparison, it was clearly observable that the RT-DETR-RK50–2 model significantly outperformed other comparative models across various complex scenarios.

In high aphid density scenes, RT-DETR-RK50–2 demonstrated superior detection and classification capabilities compared to other models. While YOLO series models exhibited classification errors and detection omissions, the RT-DETRs series presented more precise results and higher confidence scores, consistent with their excellent mAP metrics. This precision advantage stems from RT-DETRs’ architecture prioritizing detection accuracy over speed. Although RT-DETR-R50 performed adequately in specific scenarios, it showed significantly reduced stability in complex backgrounds and dense aphid regions, missing numerous targets as evidenced in row 2 of the figure. Quantitatively, RT-DETR-RK50–2 maintained consistently high confidence scores (mostly above 0.85) while comparative models showed lower or unstable confidence under similar conditions, directly validating the KAN module’s effectiveness in enhancing feature extraction depth and representational capabilities.

**Figure 10** confusion matrices revealed significant model performance differences in aphid behavior recognition. YOLO series models exhibited high Honeydew misclassification rates (YOLOv10x: 52.0%, YOLOv11-L: 57.0%, YOLOv12-M: 43.0%), indicating fundamental limitations with small targets despite their speed advantages. In contrast, RT-DETR-R50–2 achieved 86.0% Honeydew detection accuracy, far surpassing YOLO’s best performance. This critical advantage in detecting Honeydew, a key indicator of aphid damage severity, demonstrated important practical value.

**Figure 11** subplots further confirmed these differences through training dynamics. In (b), RK50–2 demonstrated optimal convergence with the lowest final loss values (~0.05) and faster convergence than R18 and R50 baselines. In (c), YOLO models stabilized at significantly higher loss levels (0.9-1.7) despite rapid initial descent. These patterns validated RT-DETR’s inherent advantages in fine-grained detection tasks, with the KAN module further enhancing performance.

Integrating the visual results from **Figure 12** with the quantitative analysis data from **Table 4**, it was concluded that RT-DETR-RK50–2 achieved optimal performance balance in aphid behavior detection tasks, not only reaching the highest mAP50 metric of 0.88 in Honeydew detection, but also maintaining consistent excellent performance across other behavioral categories. This comprehensive and robust detection capability made it particularly suitable for deployment in aphid behavior monitoring systems in real agricultural environments, providing reliable and precise phenotypic analysis technical support to accelerate the breeding process of resistant crop varieties.

**Table 4:** Detection results compared with state-of-the-art models.

Model	Backbone	mAP_0.5_ (CL)	mAP_0.5_ (LF)	mAP_0.5_ (HE)	mAP_0.5_ (All)	Params (M)	GFLOPs (G)	Latency (Ms, bs=1)
SSD	VGG	0.914	0.765	0.453	0.711	26.2	62.7	–
Faster-R CNN	Resnet50	0.876	0.681	0.131	0.563	137.0	370.210	–
YOLOv5-N	CSPDarknet-53	**0.883**	0.826	0.436	0.715	1.76	4.1	4.2 ± 0.4
YOLOv5-S	CSPDarknet-53	0.874	**0.835**	0.415	0.708	7.0	15.8	4.5 ± 0.8
YOLOv5-M	CSPDarknet-53	0.864	0.806	0.505	0.725	20.8	47.9	5.1 ± 0.3
YOLOv5-L	CSPDarknet-53	0.849	0.803	0.493	0.715	46.1	107.7	5.8 ± 0.6
YOLOv5-X	CSPDarknet-53	0.879	0.825	**0.575**	**0.760**	86.2	203.8	8.8 ± 0.9
YOLOv8-N	Darknet-53	0.846	0.792	0.113	0.584	3.0	8.1	6.0 ± 0.2
YOLOv8-S	Darknet-53	0.853	0.813	**0.257**	0.641	11.1	28.4	5.9 ± 0.5
YOLOv8-M	Darknet-53	**0.864**	0.831	0.232	**0.642**	25.8	78.7	6.9 ± 0.7
YOLOv8-L	Darknet-53	0.859	**0.833**	0.185	0.626	43.6	164.8	7.9 ± 0.1
YOLOv8-X	Darknet-53	0.847	0.816	0.253	0.639	68.1	257.4	7.8 ± 0.4
YOLOv10-N	En-CSPNet	0.844	0.672	0.344	0.672	2.7	8.2	6.7 ± 0.8
YOLOv10-S	En-CSPNet	**0.870**	0.838	0.558	0.755	8.0	24.5	7.3 ± 0.3
YOLOv10-M	En-CSPNet	0.857	**0.849**	0.467	0.724	16.4	63.4	9.4 ± 0.6
YOLOv10-L	En-CSPNet	0.847	0.825	0.650	0.774	25.7	126.4	9.9 ± 0.9
YOLOv10-X	En-CSPNet	0.853	0.839	**0.728**	**0.807**	31.5	169.8	9.4 ± 0.5
YOLOv11-N	–	**0.871**	**0.855**	0.422	0.716	2.5	6.3	7.0 ± 0.7
YOLOv11-S	–	0.867	0.843	0.654	0.788	9.4	21.3	7.3 ± 0.1
YOLOv11-M	–	0.836	0.765	0.765	0.814	20.0	67.7	7.6 ± 0.4
YOLOv11-L	–	0.851	0.830	**0.771**	**0.817**	25.3	86.6	12.5 ± 0.8
YOLOv11-X	–	0.841	0.825	**0.771**	0.812	56.8	194.4	12.4 ± 0.3
YOLOv12-N	–	0.856	0.826	0.526	0.736	2.5	6.3	4.3 ± 0.6
YOLOv12-S	–	**0.875**	0.832	0.554	0.754	9.2	21.2	3.8 ± 0.9
YOLOv12-M	–	0.856	**0.845**	0.739	**0.813**	20.1	67.1	3.5 ± 0.2
YOLOv12-L	–	0.853	0.839	0.716	0.803	26.3	88.6	5.1 ± 0.5
YOLOv12-X	–	0.849	0.838	**0.740**	0.809	59.0	198.5	7.8 ± 0.7
RT-DETR-L	HGNetv2	0.842	0.833	0.718	0.798	31.9	103.5	25.0 ± 0.1
RT-DETR-X	HGNetv2	0.825	0.819	0.808	0.817	65.4	222.5	28.0 ± 0.4
RT-DETR-R18 [7]	R18	0.852	**0.834**	0.799	0.828	19.8	57.0	**15.9 ± 0.8**
RT-DETR-P2	R18	0.854	0.822	0.765	0.814	18.6	78.2	17.1 ± 0.3
RT-DETR-R50 [7]	R50	0.844	0.818	0.798	0.820	41.9	128.6	24.6 ± 0.2
OurS	**RK50**	**0.855**	**0.832**	**0.859**	**0.849**	**84.4**	**129.6**	**27.2 ± 0.7**

The best performers in each group are highlighted in bold.

### 3.4 Model robustness analysis with aphid density and brightness conditions

**Figure 13:**
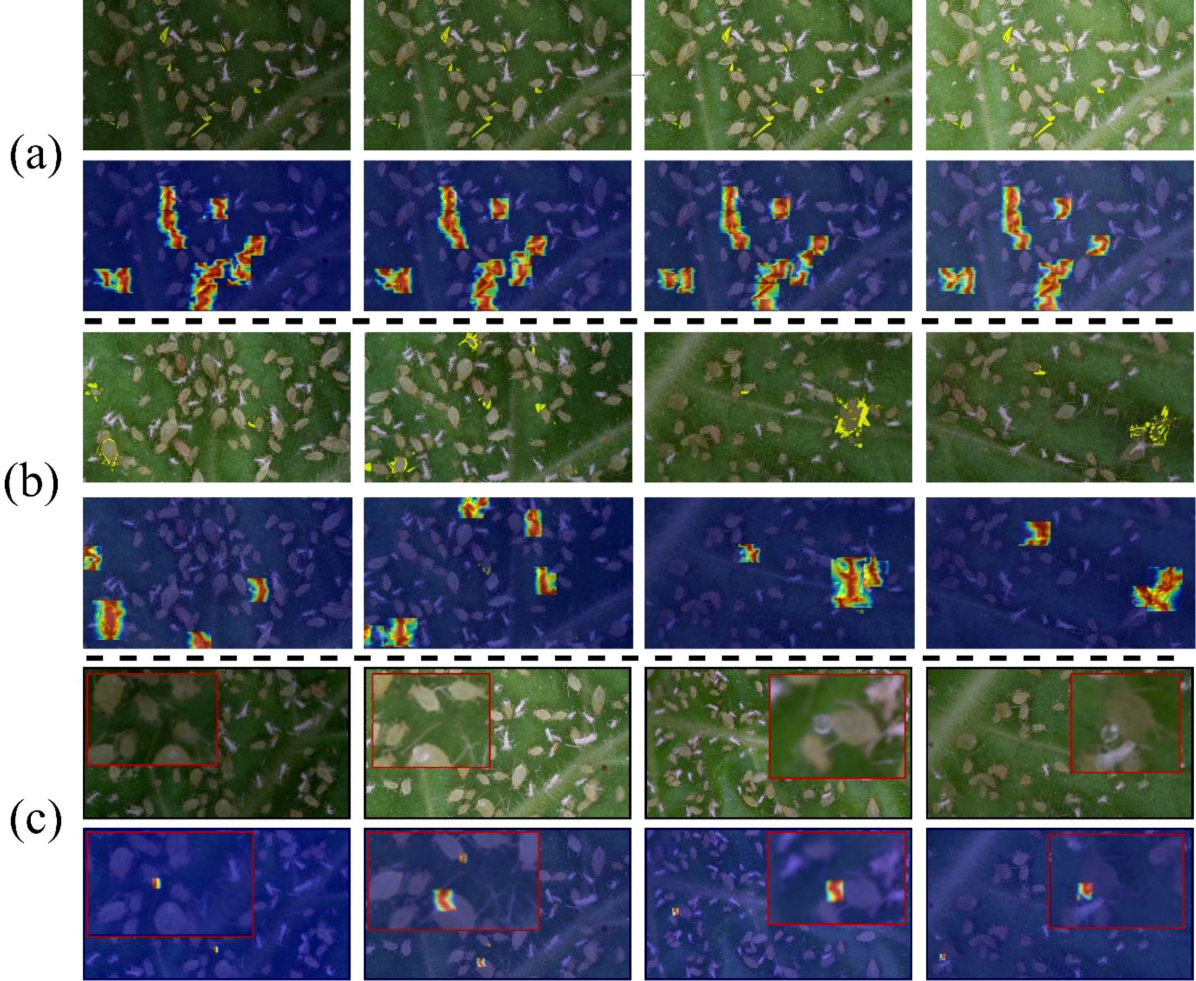
Grad-CAM++ visualization results under varying lighting conditions and Aphid densities: **(a)** Different lighting only, **(b)** Different density only, **(c)** HE target detection effect under different lighting and density.

To further evaluate the model’s performance in this study, we conducted additional visualization experiments using Grad-CAM++ to generate heatmaps, analyzing the model’s behavior under different lighting conditions and aphid densities (**Figure 13**). Specifically, experiment group (a) simulated the impact of varying brightness levels, employing brightness gains of -80, -40, 0, and 40 in this study. Experiment group (b) assessed the model’s performance across different aphid densities in this study. Experiment group (c) focused on the HE category, testing it under various combinations of brightness and density in this study.

The experimental results are shown in the figure. In group (a), the heatmap activation region was largest and most concentrated under normal brightness (brightness gain of 0), indicating the best detection performance in this study. As brightness increased or decreased, the detection performance slightly declined, but the overall difference was small, demonstrating that the RT-DETR-RK50 model exhibits good robustness to brightness variations in this study. In group (b), the model could normally detect most targets when the aphid density was low in this study. However, in high-density scenarios, one instance of missed detection occurred, indicating a limitation of the model in high-density detection in this study. In group (c), although the model could still detect targets under high density and low brightness conditions in this study, the heatmap activation region was smaller. This may be because, in high-density states, the proportion of HE target categories in the total image pixels is further reduced, making it more difficult for the model to recognize them in this study.

### 3.5 Multi-phase analysis of aphid honeydew excreting behavior detection results

**Figure 14:**
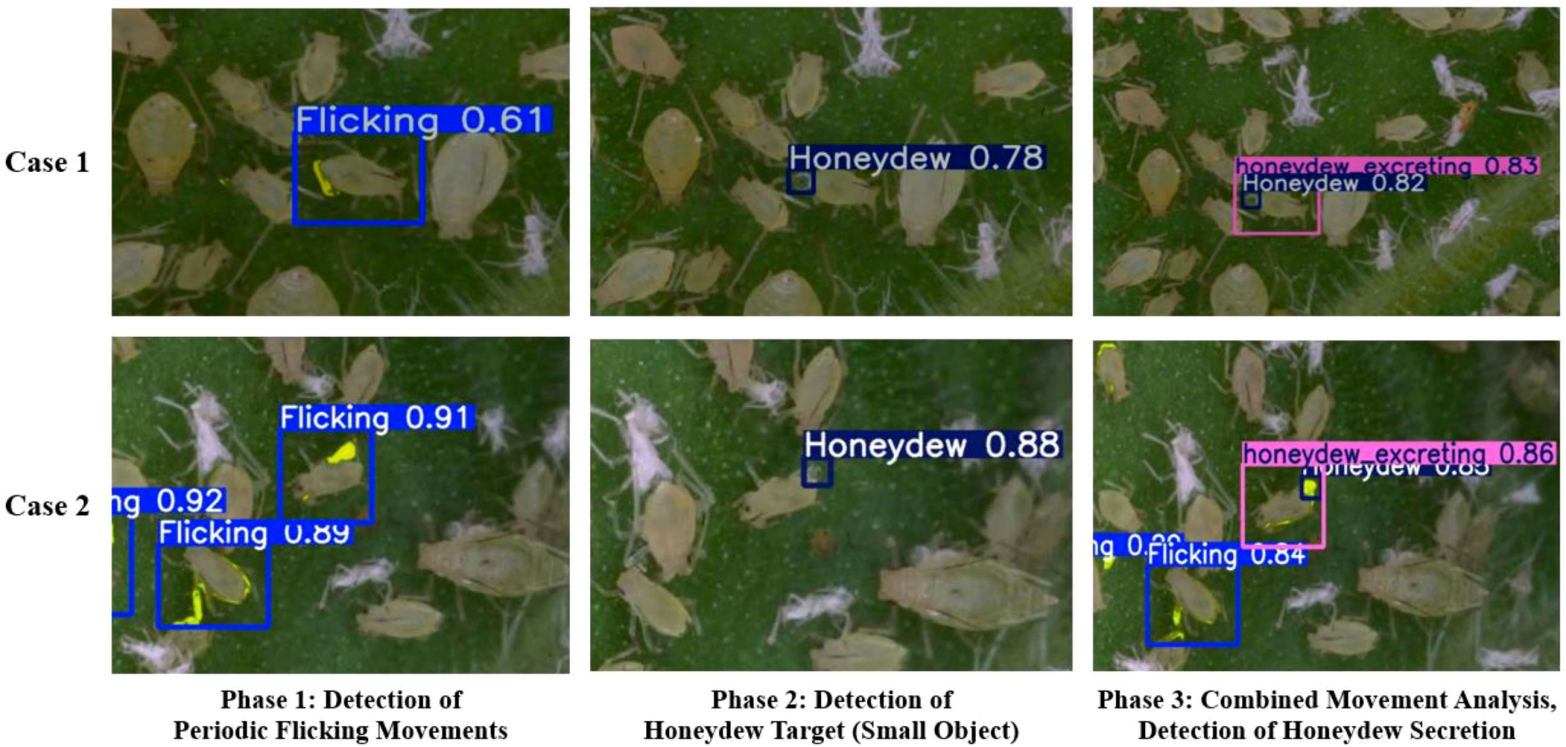
Stage-wise honeydew excreting detection pipeline and results.

As illustrated in **Figure 14**, the cross-frame detection method described in Section 2.6 successfully decomposed aphid excretion behavior into three distinct detection phases. The experimental results demonstrated the system’s capability to identify complex behavioral patterns through sequential analysis.

In Phase 1, the system detected periodic LF movements with confidence scores of 0.61 in Case 1 and 0.89-0.92 in Case 2. Blue bounding boxes effectively localized these characteristic aphid leg movements during excretion. Phase 2 focused on identifying small honeydew droplets, achieving confidence scores of 0.78 in Case 1 and 0.88 in Case 2. The blue bounding boxes in the center column precisely marked these high-reflectivity droplets on the plant substrate. In Phase 3, the system integrated temporal correlations between LF and honeydew presence, yielding honeydew excreting detection with 0.83-0.86 confidence (purple boxes), while maintaining discrete LF detection (0.64 confidence in Case 2). The right column images demonstrate the system’s ability to differentiate between combined behavioral patterns and individual components within the same frame, showcasing the advantages of object detection in high-resolution multi-target behavior recognition.

The sequential detection results confirm that the proposed methodology successfully addresses the challenges associated with detecting aphid excretion behavior. By decomposing this complex behavior into constituent components and leveraging their temporal correlation, the system achieved precise identification of both the physical actions (LF) and the physical evidence (honeydew droplets) associated with excretion events.

The high confidence scores across all phases and cases validate the effectiveness of the cross-frame processing approach in capturing these subtle behavioral indicators of aphid population vitality. The visual evidence presented in **Figure 9** demonstrates that even small-amplitude movements and minute targets can be reliably detected and classified through the implemented hierarchical detection framework.

### 3.6 Overall system real-time end-to-end detection performance

In previous research using frame differencing methods for motion feature extraction, the motion feature extraction and deep learning algorithms were typically processed in two separate stages, resulting in poor real-time performance and cumbersome processing procedures. As introduced in Section 2.7, this research optimized the detection pipeline by implementing streaming inference that synchronizes feature extraction and detection processes, achieving real-time end-to-end detection for RT-DETRs. Performance evaluations were conducted on three top-performing models based on ResNet18, ResNet50, and the improved RK50 backbone networks. As shown in the **Table 5**, these models were tested on three GPU platforms (RTX3090, RTX4090) across various resolutions (from 480p to 1080p), measuring both inference-only speed and real-time end-to-end processing speed. The time window size was set to 10 frames. The experimental results demonstrated that the proposed RK50 model, despite having slightly lower inference speeds compared to the baseline models, significantly improved detection accuracy with an mAP50 of 0.849, showing considerable performance advantages over the baseline approaches.

**Table 5:** Real-time end-to-end detection performance.

Model	GPU	Inference speed only	Real-time end-to-end processing speed			mAP_50_ (all)
			480p	720p	1080p	
RT-DETR-R18	RTX3090	58.47	60.61	46.98	33.63	0.828
RT-DETR-R50	RTX3090	33.33	46.98	37.24	27.81	0.820
RT-DETR-RK50	RTX3090	28.16	40.72	33.59	23.73	0.849
RT-DETR-R18	RTX4090	62.80	68.71	50.69	36.45	0.828
RT-DETR-R50	RTX4090	40.65	54.44	42.69	31.65	0.820
RT-DETR-RK50	RTX4090	36.70	50.49	41.99	31.82	0.849

The architectural optimizations delivered significant performance improvements across the model lineup. At 1080p resolution, RT-DETR-RK50 maintained 31.82 fps on RTX4090, exceeding the 30 fps real-time processing threshold. Notably, the data revealed that adding computational work paradoxically increased throughput, particularly at lower resolutions where RT-DETR-RK50 on RTX4090 reached 50.49 fps for 480p and 41.99 fps for 720p. This enhancement stemmed from efficient resource utilization through workload distribution across multiple threads and CUDA streams while overlapping I/O with computation, minimizing idle GPU time and transforming sequential bottlenecks into concurrent operations.

The optimization effects were particularly pronounced on more powerful hardware, with RTX4090 configurations consistently outperforming RTX3090 counterparts by approximately 10-25%, and at 1080p resolution, the RTX4090 implementation achieved a 34% performance advantage over the RTX3090 for the same model (31.82 fps vs 23.73 fps). This demonstrated how the architecture efficiently scaled with available computational resources. These results showed that properly engineered parallel systems could enable real-time, end-to-end detection at resolutions previously considered impractical for simultaneous feature extraction and object detection, while maintaining high accuracy (RT-DETR-RK50 achieved 0.849 mAP50). Through comparative analysis of accuracy-inference speed across different models, various models could be selected for inference based on different application scenarios within the high-throughput end-to-end RT-DETRs aphid behavior detection framework, substantially expanding practical applications in high-fidelity video analysis scenarios.

### 3.7 Limitations and future works

This study achieved promising results in aphid behavior recognition, though certain limitations indicate directions for future research.

Dataset quality remains foundational for behavior recognition, with current limitations in collection constrained by breeding cycles and aphid activity patterns. The annotation quality requires enhancement as behavior categorization remains relatively broad, failing to capture the full spectrum of aphid behaviors including probing feeding, antennal movement, reproduction, and molting. Future work should develop more comprehensive behavioral datasets despite the high cost of annotation, which presents an ongoing challenge.

This study aims to address the issues of computational efficiency and field deployment readiness. Despite implementing accelerated processing and achieving real-time detection, resource utilization on the 4090 GPU remained suboptimal (power consumption: 50W/425W, memory usage: 1612MiB/24564MiB). Future research should employ more efficient acceleration techniques, reduce operational demands, and establish a systematic detection platform. To further enhance the system’s adaptability to resource-constrained environments, this study will explore several strategies.

This study initially employs high-resolution original video for feature fusion to ensure finer details are available for the 640*640 RT-DETR algorithm, thereby improving detection performance. To mitigate computational demands, frame skipping (e.g., processing only 1/4 or 1/8 of the frames) can be implemented, or time window parameters can be optimized based on target characteristics. Recognizing the trade-off between resolution and speed, utilizing lower-resolution inputs can significantly accelerate detection while reducing hardware requirements. As previously noted, the efficiency of the 4090 GPU can be further optimized through parallel computing techniques. Furthermore, the feature fusion method proposed in this study enables the flexible selection of alternative detection models based on the available hardware. For instance, lightweight algorithms such as YOLO or DINO can be employed, accepting a slight reduction in accuracy to achieve lower hardware costs and enable deployment in resource-limited settings. Future work will systematically evaluate these adaptations to ensure optimal performance across diverse deployment scenarios.

While the current method performs well for short-term behavior recognition, integration with algorithms like Long Short-Term Memory (LSTM) (Hochreiter and Schmidhuber, 1997) or more efficient DETR extensions could maintain real-time advantages. Implementation of tracking algorithms such as BoT-SORT (Aharon et al., 2022) and ByteTrack (Zhang et al., 2022) could enhance the development of a reliable behavior recognition platform.

Future research will focus on several key areas to enhance the generalization validation of the model. First, this research plans to collaborate with multiple agricultural pest monitoring agencies to collect behavioral videos of species such as cotton aphids and wheat aphids on wheat and cotton crops. Domain adaptation techniques will then be employed to optimize the model’s robustness across different crop backgrounds and further verify its cross-scenario applicability. Second, while the current motion feature extraction method has proven effective for large-scale behaviors (CL, LF), its performance with fine-grained behaviors requires further investigation using more precisely categorized datasets. Finally, the applicability of this research methodology is not limited to aphids but can also be extended to similar insects such as whiteflies. A future research direction is to develop multi-category insect behavior recognition datasets to verify broader generalizability across different species.

## 4 Conclusion

This study established the first aphid excretion behavior dataset comprising three behaviors: CL, LF, and HE. To address the challenge of detecting the subtle HE behavior, this study innovatively decomposed this fine-grained behavior into a three-stage sequence: Flicking-Honeydew Formation-Honeydew Excreting. Through sequential detection, the system successfully captured the temporal correlation between physical actions (LF) and physical evidence (honeydew droplets) in excretion events, overcoming the limitations of traditional methods in identifying minute behaviors. To meet the demand for extracting refined motion features from 1080p high-resolution videos, this study developed a Rapid Adaptive Motion-Feature Fusion algorithm utilizing GPU multi-threading acceleration to achieve real-time processing (45fps), providing high-granularity spatiotemporal motion information for subsequent deep learning models.

Based on this feature extraction framework, this study developed the RT-DETR-RK50 algorithm by integrating KAN networks into ResNet50 and introducing spline-based adaptive non-linear activation functions within Block structures, significantly enhancing feature extraction capabilities. Through comprehensive ablation studies, this study systematically investigated how improved RK50 Block components affected feature representation redundancy, hierarchy organization, and detection accuracy, determining the optimal deployment strategy. Results demonstrated that the method achieved 84.9% mAP50, a 2.9% improvement over the original ResNet50, outperforming existing state-of-the-art algorithms.

For deployment in practical application scenarios, this study thoroughly optimized the RT-DETR architecture with motion feature cross-frame streaming inference, detection result delay interpolation, and composite behavior merging logic, achieving end-to-end real-time detection from input video stream to detection results at 31.82fps with 1080p high-fidelity resolution. This significantly reduced the complexity and intermediate storage resource consumption from feature processing to detection processing stages, substantially enhancing the system’s application scalability. This aphid behavior detection solution can be extended to monitor similar insects such as whiteflies, providing high-throughput non-contact monitoring support for pest control, resistance breeding, and crop protection. Future research could further expand behavior categories to include reproduction, molting, and sap-feeding, while seeking optimal balance between computational accuracy and detection speed.

## Data Availability

The datasets presented in this study can be found in online repositories. The names of the repository/repositories and accession number(s) can be found in the article/**Supplementary Material**.
