# Supplementary material for: High-throughput end-to-end aphid honeydew excretion behavior recognition method based on rapid adaptive motion-feature fusion
Source: Front Plant Sci. 2025 Jul 7;16:1609222. doi: 10.3389/fpls.2025.1609222 (PMC12277367; doi:10.3389/fpls.2025.1609222)
Supplement: Supplementary file 1 [file Table1.docx]

**Appendix A. Algorithm 1**

| **Algorithm 1**: Real-Time Motion Enhanced Object Detection |
| --- |
| **Input**: Video frame sequence **V**, **RTDETR** model **M**, color thresholds [**Lc**, **Uc**]  **Output**: Enhanced video with specialized detection labels and motion visualization  // Initialize components and optimizations  1: Initialize **RTDETR** model with **TorchScript** optimization and prewarming  2: Initialize frame queues **Qgray**, **Qmask**, **Qcolor** of size **TimeWindow** = 10  3: Initialize detection history **Hdetect** = [] // For temporal label fusion  4: Initialize even/odd frame caches **Ceven** = **Codd** = ∅, missing counters **Me** = **Mo** = 0  5: Initialize **CUDA** streams **S**[3] and memory pools for efficient GPU processing  6: Initialize parallel processing queues **Qframe**, **Qresult** with thread synchronization  // Parallel video processing pipeline  7: Start **reader_thread**(**input_video**, **Qframe**, **done_event**)  8: Start **processor_thread**(**Qframe**, **Qresult**, **done_event**)  9: Start **writer_thread**(**Qresult**, **output_video**, **done_event**)  // Frame processing in processor_thread  10: **For** each batch of frames:  11: **For** each frame **f** in batch:  // Motion preprocessing (runs on dedicated **CUDA** stream)  12: Move frame to GPU: **fGPU** = **f**.to(**device**)  13: Compute color mask using vectorized operations: **mask** = **getColor_batch**(**fGPU**, **Lc**, **Uc**)  14: Convert to grayscale using GPU: **gray** = mean(**fGPU**, dim=2)  15: Add **gray**.reshape(-1) to **Qgray**, **mask**.reshape(-1) to **Qmask**, **fGPU** to **Qcolor**  16: **global_frame_idx** += 1  17: **is_even_frame** ← (**global_frame_idx** % 2 == 0)  // Motion detection (every 2 frames and when time window is full)  18: **If** **is_even_frame** **AND Qgray** is full:  19: Compute frame differences with optimized GPU operations  20: Compute threshold: **Diffthresh** = mean(**Diff**) + 3·std(**Diff**)  21: Compute center of gravity using GPU **FFT**  22: Apply GPU-accelerated denoising with pre-computed Gaussian kernel  23: Create enhanced motion visualization with alpha blending and color gradients  24: **fenhanced** ← **motion_visualization**  25: **Else**: **fenhanced** ← **f**  // Batch inference with optimized model  26: **results** ← **M**.predict(batch_of_**fenhanced**) with **inference_mode** and mixed precision  // Post-processing for each result  27: **For** each result **r** in **results**:  28: Update class names mapping if needed  29: Extract current detected labels for temporal fusion  30: Update **Hdetect** with timestamp and labels, removing entries older than 2 seconds  // Handle even/odd frame specific object caching with missing counters  31: **If** **is_even_frame**:  32: Extract **abc_objects** (Crawlling, Flicking) and update **Ceven if** objects found  33: **If** no **abc_objects** found: **Me** += 1, use cached **Ceven if** **Me** < 2  34: Extract **ball_objects** (Honeydew) and update **Codd if** objects found  35: **If** no **ball_objects** found: **Mo** += 1, use cached **Codd if** **Mo** < 2  36: **Else**:  37: Extract **ball_objects** (Honeydew) and update **Codd if** objects found  38: **If** no **ball_objects** found: **Mo** += 1, use cached **Codd if Mo** < 2  39: Extract **abc_objects** (Crawlling, Flicking) and update **Ceven if** objects found  40: **If** no **abc_objects** found: **Me** += 1, use cached **Ceven if Me** < 2  // Reset caches if missing too many frames  41: **If Me** >= 2: **Ceven** = ∅  42: **If Mo** >= 2: **Codd** = ∅  // Process honeydew secretion detection  43: **If** **abc_objects** **and ball_objects**:  44: **For** each **behavior_box** in **abc_objects** (classes Crawling, Flicking):  **Algorithm 1**: Real-Time Motion Enhanced Object Detection |
| 45: **For** each ball in **ball_objects**:  46: **If** ball center is inside **behavior_box**:  47: Replace **behavior_box** class with "Honeydew Excreting" class  48: Mark ball as processed  49: Merge remaining **abc_objects**, **ball_objects** into final detections  // Handle specialized label combinations  50: **If** "Flicking" and "Crawlling" both in Hdetect within 2-second window:  51: Replace these labels with "Honeydew Excreting" in current result  52: Plot detections on frame and add to **Qresult**  // Clean up resources and return statistics  53: Wait for all threads to complete  54: Release video resources and CUDA memory  55: **Return** processing statistics  // Complexity Analysis  // Time: O(n·(w·s·log(s) + b·d)), where n=frames, w=window size, s=pixels/frame, b=batch size, d=detection complexity  // Space: O(w·s + b·f + p), where f=frame size, p=GPU memory pool size |
